# Tumor cell spheroid-induced suppression of primary human cytotoxic T cells as a scalable *in vitro* model of exhaustion

**DOI:** 10.1093/immadv/ltaf023

**Published:** 2025-06-11

**Authors:** Amal Alsubaiti, Hanin Alamir, Lan Huynh, Tressan Grant, Abdullah Aljohani, Po Han Chou, Yiwei Shi, Maryam Alismail, Lydia R Mason, Andrew Herman, John S Bridgeman, Christopher J Holland, Christoph Wülfing

**Affiliations:** School of Cellular and Molecular Medicine, University of Bristol, Bristol BS8 1TD, United Kingdom; School of Cellular and Molecular Medicine, University of Bristol, Bristol BS8 1TD, United Kingdom; School of Cellular and Molecular Medicine, University of Bristol, Bristol BS8 1TD, United Kingdom; School of Cellular and Molecular Medicine, University of Bristol, Bristol BS8 1TD, United Kingdom; Department of Life Sciences, University of Bath, Bath BA2 7AY, United Kingdom; School of Cellular and Molecular Medicine, University of Bristol, Bristol BS8 1TD, United Kingdom; School of Cellular and Molecular Medicine, University of Bristol, Bristol BS8 1TD, United Kingdom; School of Cellular and Molecular Medicine, University of Bristol, Bristol BS8 1TD, United Kingdom; School of Cellular and Molecular Medicine, University of Bristol, Bristol BS8 1TD, United Kingdom; School of Cellular and Molecular Medicine, University of Bristol, Bristol BS8 1TD, United Kingdom; School of Cellular and Molecular Medicine, University of Bristol, Bristol BS8 1TD, United Kingdom; Department of Research, Instil Bio, Dallas, TX 75219, United States; Immunocore Ltd., Abingdon, OX14 4RY, United Kingdom; School of Cellular and Molecular Medicine, University of Bristol, Bristol BS8 1TD, United Kingdom

**Keywords:** human, T cell, adoptive cell transfer

## Abstract

**Background:**

Cytotoxic T lymphocytes (CTL) are key effectors in the antitumor immune response. However, their function is commonly suppressed in tumors in the form of exhausted CTL. Understanding mechanisms of suppression and of therapeutics to overcome them is of substantial basic and translational importance yet hindered by limited access to large numbers of exhausted CTL in vitro.

**Methods:**

Here we use three-dimensional tissue culture to generate primary human CTL with suppressed function. Using functional assays, a 21-antibody flow cytometry panel and determination of calcium signaling and CTL tumor cell couple maintenance, we have characterized their phenotype.

**Results:**

We show that these cells closely resemble exhausted CTL from tumors. For a better understanding of in vitro human primary CTL as key tools in therapeutic development, before and after induction of suppression, we have determined the dependence of CTL function on methodology of generation, antigen dose, and affinity across two T–cell receptors and multiple tumor cell lines. As a further determination of their phenotype, we have investigated the morphology and subcellular F-actin distributions of CTL as key regulators of effector function. Primary human CTL formed cell couples with tumor target cells even in the absence of antigen. Yet, the gradual stabilization of such cell couples was associated with increasing CTL effector function. Induction of suppression substantially destabilized CTL tumor cell couples.

**Conclusion:**

This comprehensive characterization of the phenotype of in vitro primary human CTL, including a suppressed state, should facilitate their use in basic research, the development of CTL-targeting therapeutics and the determination of their mechanism of action.

## Graphical Abstract

**Graphical Abstract:**
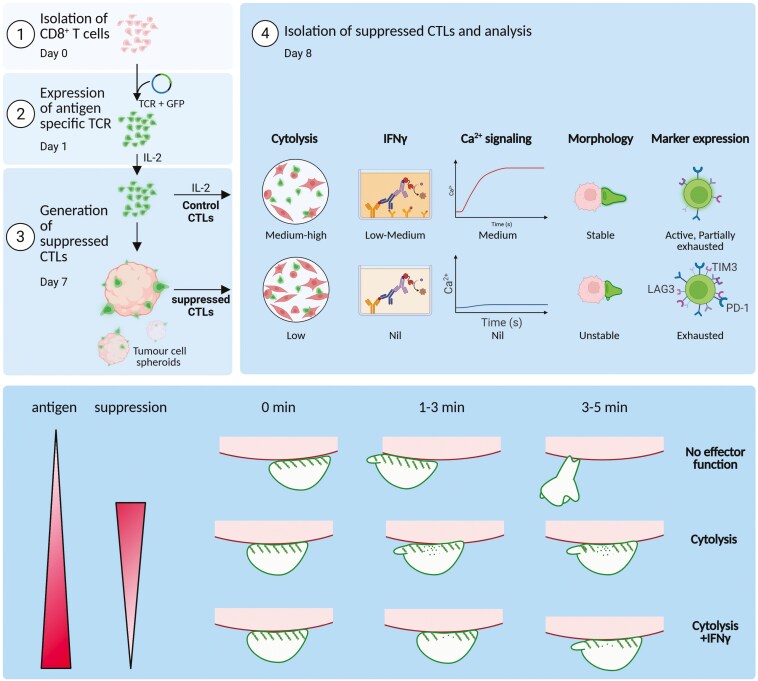

## Introduction

Cancer is frequently associated with an antitumor immune response that involves most immune cell types. CD8^+^ cytotoxic T lymphocytes (CTL) have the inherent ability to kill tumor target cells, yet this ability is commonly suppressed in the tumor microenvironment. Omics-based analyses of patient samples have established that CTL suppression consists of multiple progressive CTL states from precursor-exhausted to terminally exhausted CTL [1–4]. Understanding the mechanisms of CTL suppression and how therapeutics can overcome them is of wide interest. Such research greatly benefits from efficient, scalable access to suppressed CTL. Mouse models provide access to suppressed CTL and have greatly contributed to the understanding of antitumor immune responses [5]. However, tumor development is commonly accelerated in mice, keeping mice under specific pathogen-free conditions as universally done alters key aspects of immune function [6] and using mice in therapeutic development requires the generation of matched murine and human compounds. Humanized mice are available yet require substantial genetic engineering [7]. Therefore, effective access to human-suppressed CTL is of substantial utility. Patient-derived organoids that retain some immune function or can shape CTL function are now available [8, 9]. They can provide an in vitro model of the interaction of the entire immune system with a tumor, yet application at scale is difficult. Organ-on-chip models offer the potential of scale [10, 11], yet their validation against in vivo biology can be challenging. Here we present a scalable experimental approach to generate primary human-suppressed CTL that closely resembles tumor-infiltrating CTL.

In previous murine work, we have shown that the phenotype of CTL after interaction with three-dimensional tumor cell spheroids in the presence of antigen closely resembles the phenotype of the same CTL interacting with the same tumor cells grown subcutaneously in vivo [12]. Here we have transferred this strategy to human cells in two steps, a characterization of primary human CTL expressing a defined TCR and the interaction of such CTL with tumor cell spheroids to induce suppression. Human CTL were grown from peripheral blood mononuclear cells (PBMC) and lentivirally transduced to express the 1G4 or MEL5 T cell receptor (TCR) recognizing peptides derived from the tumor-associated antigen New York esophageal squamous cell carcinoma 1 (NY-ESO-1) or melanoma antigen A(Melan-A)/melanoma antigen recognized by T cells 1 (MART-1), respectively [13, 14]. We have characterized cytolysis and IFNγ secretion as a function of the methodology of CTL generation and across peptide concentrations and affinities in response to Mel624 or A375 melanoma and NCI-H1755 non-small cell lung carcinoma cells. For effective killing, CTL need to undergo a series of subcellular polarization steps that cumulate in the release of lytic granules [12, 15]. With increasing stimulus strength gradual stabilization of CTL tumor cell couples was associated with increasing CTL effector function. Interaction of these human CTL with tumor cell spheroids induced further suppression, as evident in substantially reduced effector function, impaired subcellular polarization, loss of calcium signaling, and a flow cytometry phenotype that closely resembles exhausted CTL from tumors as determined with a 21-antibody panel. This work thus provides at-scale access to primary human-suppressed CTL of an extensively characterized phenotype. These cells allow for efficient and physiological investigation of mechanisms of CTL suppression and mechanisms of action of therapeutics, as already initiated [16].

## Materials and methods

### Human blood samples

Blood buffy coats from anonymous donors were purchased from NHS-BT with human work approved by the London-Riverside Research Ethics Committee under reference number 20/PR/0763.

### Human cell culture

Human A375 melanoma (RRID:CVCL_0132), Mel624 melanoma (RRID:CVCL_8054) and NCI-H1755 non-small cell lung carcinoma (RRID:CVCL_1492) cells were stably transfected to express mCherry or tdTomato using a plasmid derived from pIRES Hygro (Addgene). A375 and Mel624 cells were maintained in high glucose DMEM with 10% FBS, 2 mM Glutamine, and 1 mM pyruvate (DMEM complete medium). NCI-H1755 was maintained in RPMI1640 with 10% FBS and 2mM Glutamine (RPMI complete medium). Medium for the growth of mCherry or tdTomato transfectants was supplemented with 250 μg/ml Hygromycin. Primary human CTL were derived from blood buffy coats, lentivirally transduced for the expression of defined TCRs, and maintained in cell culture as described in the Supplementary Materials.

Isolation of tumor-infiltrating lymphocytes. Around 0.5g of freshly dissected specimen was collected from melanoma patients undergoing surgery at the UHBW Hospital. Specimens were maintained in 20 ml of MACS® Tissue Storage Solution (Miltenyi Biotec) at 2−8°C. Samples were processed within 4 hours of collection. Tumour samples were dissected into 2–4 mm pieces and enzymatically digested using the Human Tumour Dissociation Kit (Miltenyi Biotec). Samples were filtered to single-cell suspension by passing through a 70 µm cell strainer, centrifuged at 1500 rpm for 5 minutes, and resuspended in PBS to 1 × 10^6^ cells/ml.

### Spheroids and SILs

A375 tdTomato or Mel624 tdTomato cells were resuspended at a concentration of 1 × 10^5^ cells/ml, mixed with Matrigel (Corning) at 4°C, seeded in a 24-well plate at a final concentration of 500 cells per Matrigel dome, and left to solidify for 10 minutes at 37°C. 2 ml DMEM complete medium was added to each well and cells were incubated at 37°C for 11 days as the default and up to 17 days in the characterization of spheroid properties. To determine spheroid properties, bright field images were acquired and the area of the spheroid cross-section, spheroid circularity, and roundness were measured using the particle analysis function of ImageJ/Fiji. NCI-H1755 cells were prepared in RPMI complete medium supplemented with 2.5% Matrigel. The cells were seeded in a 96-well round-bottom plate precoated with 1% agarose at a concentration of 2000 cells per well. The plate was then centrifuged at 1000 rpm for 5 minutes to encourage cells to cluster. Cells were maintained in culture for 5–8 days.

For the generation of spheroid-infiltrating lymphocytes (SIL), each Matrigel dome was washed twice in PBS and incubated for 1 hour with 1 ml of Cell Recovery Solution (Corning). Spheroids were collected in a 15-ml Falcon tube and pulsed with NY-ESO-1 peptide at a final concentration of 2 μg/ml for 1 hour or left unpulsed. Spheroids were re-embedded in Matrigel together with 5 × 10^6^ 1G4 CTL per Matrigel dome. Matrigel domes were dissolved for analysis of spheroid-infiltrating T cells after 16 hours: Spheroids were washed twice in PBS and incubated with 1ml of Cell Recovery Solution (Corning). Spheroids were collected, washed through a 40 μm sieve, and then disaggregated to retrieve T cells in 500 μl of imaging buffer for immediate FACS sorting.

Spheroid imaging is described in the Supplementary Materials.

### Cytolysis and IFNg secretion

For imaging-based cytotoxicity assays, the IncuCyte™ Live Cell analysis system (Essen Bioscience) was used to quantify target cell death. 1 × 10^6^ Mel624, A375 or NCI-H1755 cells transfected to express the fluorescent protein mCherry or tdTomato were either untreated or pulsed for 1h with the indicated concentration of HLA-A*02:01 NY-ESO-1_157-165_ (SLLMWITQC), HLA-A*02:01 MART-1_26-35_ with the A27L mutation (‘ELA’) (ELAGIGILTV) or HLA-A*02:01 MART-1_26-35_ with the E26F, A28T double mutation (‘FAT’)(FATGIGILTV). Cells were suspended in 5 ml Fluorobrite medium (Thermo Fisher) with 10% FBS, 2 mM L-glutamine, 50 µM 2-mercaptoethanol to a concentration of 15 000 cells/50 μl. Cells were plated in a 384-well Perkin-Elmer plastic-bottomed plate and incubated for 4 hours to adhere. 40,000 CTL that had been FACS sorted for indicated expression of GFP were added per well to the plate in 50 μl Fluorobrite medium, yielding a 4:1 effector to target ratio. Images were taken every 15 min for 14 hours at 1600 ms exposure using a 10× (NA = 0.3) lens. The total red object (mCherry/tdTomato target cell) area (µm^2^/well) was quantified at each time point. A change in red area was determined as the linear gradient of the red object data at its steepest part for 6 hours. The CTL killing rate was calculated as the difference in such change in red area in the presence and absence (to account for tumor cell proliferation) of CTL in the same 6 hours time window.

To determine IFN**g** secretion, supernatants at the end of the imaging-based cytolysis assays were collected and frozen. The human IFN**g** OptEIA ELISA Kits (BD Biosciences) was used according to the manufacturer’s instructions.

### Imaging of CTL and SIL

Prior to imaging, CTL and SIL were resuspended in ‘imaging buffer’ (10% FBS in PBS with 1 mM CaCl_2_ and 0.5 mM MgCl_2_). As target cells, 1 × 10^6^ Mel624 or A375 melanoma cells were pulsed with the indicated peptide (as listed above) at a final concentration of 2 µg/mL for 1 hour or left unpulsed. Glass bottomed, 384-well optical imaging plates (Brooks Life Science Systems) were used for all imaging experiments. Imaging of F-actin distributions and CTL morphology was done at 37^o^C using an Olympus IXplore SpinSR confocal system incorporating a Yokogawa CSU-W1 SoRa spinning disk with a 60× oil-immersion lens (NA = 1.5). Every 20 seconds for 15 minutes, a z-stack of 53 GFP images (0.25 µm z-spacing) was acquired, as well as a single, mid-plane differential interference contrast (DIC) reference image.

For imaging the elevation of the cytoplasmic calcium concentration, 1G4 T cells were incubated with 2 µM Fura-2 AM (Molecular Probes) for 30 minutes at room temperature in the imaging buffer and washed thereafter. 1G4 T cells were activated with Mel624 APCs as described above and three images were acquired every 10 s for 15 minutes, one bright field image, one fluorescence image with excitation at 340 nm, and one fluorescence image with excitation at 380nm. Imaging data were acquired at 37^o^C using a 40× oil objective (NA = 1.25) on a Leica DM IRBE-based wide filed system equipped with Sutter DG5 illumination and a Photometrics Coolsnap HQ2 camera.

Image analysis is described in the Supplementary Materials.

### Flow cytometry

The antibodies of the 21-color panel are listed in the Supplementary Material. 1 × 10^6^ cells were first stained with a 1:10 000 dilution of the LIVE/DEAD™ Fixable Red Dead Cell Stain (ThermoFisher) for 15–30 minutes in the dark at room temperature (RT) and washed with FACS buffer (PBS, 0.5% BSA, 2.5 mM EDTA). Fc receptors were blocked with 10 µl of a 1:20 dilution of Fc Blocker (Thermo Fisher) in the dark at RT for 10 minutes. 40 µl of biotinylated antibody was added and incubated at 4°C for 30 minutes. The sample was washed with FACS buffer and incubated with 10 µl of a 1:20 dilution of Monocyte Blocker (BioLegend) in the dark at RT for 10 minutes. 40 µl of an antibody master mix against cell surface proteins as listed in the antibodies section was added for the total volume of 50 µl, incubated at 4°C for 30 minutes, and washed with FACS buffer. Cells were fixed and permeabilized using the True-Nuclear™ Transcription Factor Buffer Set (BioLegend). Cells were stained for intracellular proteins with an antibody master mix as listed in the antibodies section for 30 minutes at 4°C. Cells were washed with permeabilization buffer and resuspended in FACS buffer and kept at 4°C until the next day. For the flow cytometry data acquisition, samples were run on a four-laser Cytek® Aurora system (4L V/B/YG/R) on low to medium flow rates (15–30 µl/min) and analysed using SpectroFlo® software with autofluorescence extraction and live unmix during sample acquisition. The data for the unmixed samples were processed using the FlowJo software (v.10.10.0). Positive gates were set using fluorescence-minus-one data.

The same protocol, live/dead stain, Fc block, cell surface antibodies, permeabilization, and internal antibodies, was used for single antibody flow cytometry experiments with steps omitted as allowed by the antigen to be stained. For tetramer staining, some samples were treated with 50 nM of the tyrosine kinase inhibitor dasatinib (a gift from L. Wooldridge, U. Bristol) to enhance tetramer staining.

Cluster analysis is described in the Supplementary Materials.

## Results

### Primary human CTL expressing a transgenic TCR can kill tumor targets upon endogenous antigen presentation

The generation of suppressed primary human CTL required two steps, the generation of primary human CTL expressing a transgenic TCR and the subsequent interaction of such cells with tumor cell spheroids. To generate primary human CTL expressing a transgenic TCR, we isolated CD8^+^ T cells from buffy coats and activated these cells with beads coated with antibodies against CD3ε and CD28. We transduced these cell cultures with a human immunodeficiency virus (HIV)-derived lentivirus driving the expression of the alpha and beta chains of a transgenic TCR and GFP as a transduction marker (Supplementary Text, Figs. S1, S2). We used two TCRs. The 1G4 TCR recognizes peptides 157-165 of the tumor-associated antigen NY-ESO-1 presented by HLA-A*0201 [13]. The MEL5 TCR recognizes peptides 26-35 of the tumor-associated MART-1 also presented by HLA-A*0201 [14].

To evaluate the cytolytic function of the human primary CTL expressing a transgenic TCR, we used an imaging-based cytotoxicity assay [12]. For use in the imaging-based killing assay, all target cell lines were stably transfected to express the red fluorescent protein mCherry. Cytolysis was determined as a reduction in the red area of peptide-loaded mCherry-transfected target cells adhering to the bottom of a 384-well imaging plate. A rate of 10% red area loss per hour constitutes efficient killing [12]. As secretion of IFN**g** is a key second effector function of CTL that can be regulated differently than target cell killing, we also measured the amount of IFN**g** at the end of the imaging-based cytolysis assays in the assay supernatant. We used three target cell lines expressing HLA-A*0201, the melanoma cell lines Mel624 and A375, and the non-small cell lung carcinoma cell line NCI-H1755. Endogenous NY-ESO-1 (NCI-1755 > A375 > Mel624) and MART-1 (Mel624 > A375) expression is characterized in the Supplementary Material (Supplementary Text, Fig. S3). The dependence of cytolysis on the methodology of CTL in vitro culture is characterized in the Supplementary Material (Supplementary Text, Figs. S1, 2, 4). Of importance for the interpretation of the subsequent data, the lentiviral expression of the recombinant TCRs as stabilized with an additional disulfide bridge in the constant domains, the default for the experiments here, was inefficient (Supplementary Figs. S1, S2). The use of murine constant domains to increase recombinant TCR expression is described below.

**Figure 1.**
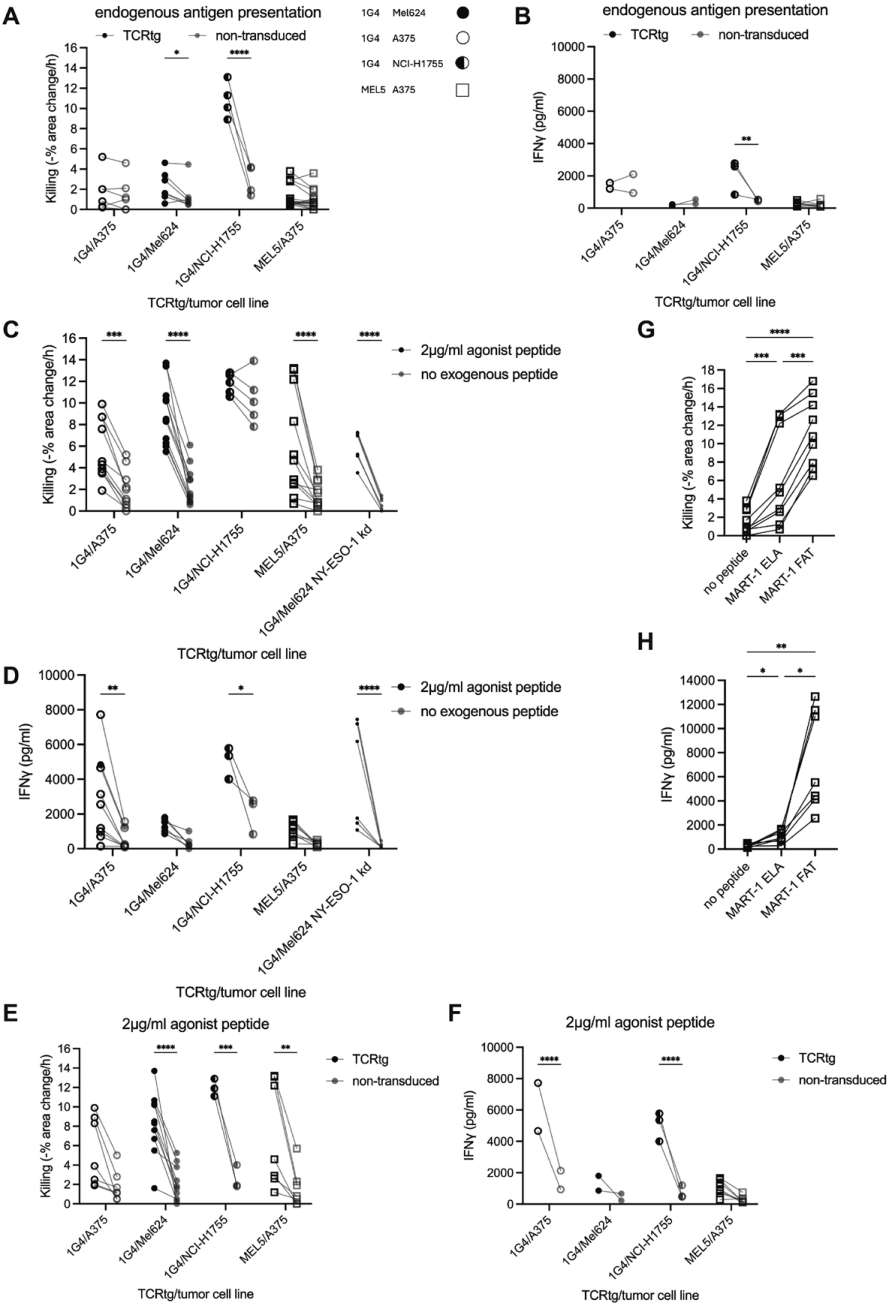
Agonist peptide amount and affinity determine the function of primary human CTL expressing a transgenic TCR. A. Killing of A375, Mel624, or NCI-H1755 tumor target cells by 1G4 or MEL5 CTL in comparison to CTL with an endogenous TCR repertoire (‘nontransduced’) and symbols used across all figures for the interaction of CTL transduced to express a transgenic TCR, 1G4 or MEL5, with indicated tumor target cell lines. 33 independent experiments. Statistical significance was determined by paired Two-way ANOVA. B. IFNg amounts in supernatants of A375, Mel624, or NCI-H1755 tumor target cells after 16h of interaction with 1G4 or MEL5 CTL in comparison to CTL with an endogenous TCR repertoire (‘nontransduced’). Sixteen independent experiments. Statistical significance was determined by paired Two-way ANOVA. C. Killing of A375, Mel624, Mel624 NY-ESO-1 kd or NCI-H1755 tumor target cells incubated with or without 2µg/ml NY-ESO-1 or ELA MART-1 agonist peptide by 1G4 or MEL5 CTL. Forty two independent experiments. Statistical significance was determined by paired Two-way ANOVA. D. IFNg amounts in supernatants of A375, Mel624, Mel624 NY-ESO-1 ko or NCI-H1755 tumor target cells incubated with or without 2µg/ml NY-ESO-1 or ELA MART-1 agonist peptide after 16 hours interaction with 1G4 or MEL5 CTL. Thirty one independent experiments. Statistical significance was determined by paired Two-way ANOVA. E Killing of A375, Mel624, or NCI-H1755 tumor target cells incubated with 2µg/ml NY-ESO-1 or ELA MART-1 agonist peptide by 1G4 or MEL5 CTL in comparison to CTL with an endogenous TCR repertoire (‘nontransduced’). Twenty seven independent experiments. Statistical significance was determined by paired Two-way ANOVA. F. IFNg amounts in supernatants of A375, Mel624, or NCI-H1755 tumor target cells incubated with 2 µg/ml NY-ESO-1 or ELA MART-1 agonist peptide after 16 hours interaction with 1G4 or MEL5 CTL in comparison to CTL with an endogenous TCR repertoire (‘nontransduced’). Fourteen independent experiments. Statistical significance was determined by paired Two-way ANOVA. G. Killing of A375 tumor target cells incubated with or without 2 µg/ml ELA or FAT MART-1 agonist peptide by MEL5 CTL. Nine independent experiments. Statistical significance was determined by paired One-way ANOVA. H. IFNg amounts in supernatants of A375 tumor target cells incubated with or without 2 µg/ml ELA or FAT MART-1 agonist peptide after 16 hours interaction with MEL5 CTL. Seven independent experiments. Statistical significance was determined by paired One-way ANOVA. * *P* < .05, ** *P* < .01, *** *P* < .001, **** *P* < .0001.

To determine whether expression of a transgenic TCR in human primary CTL could confer antigen specificity in response to endogenous antigen presentation, we compared the ability of CTL transduced to express a transgenic TCR to kill tumor target cells in the absence of exogenous agonist peptide to that of primary human CTL expressing only an endogenous TCR repertoire (Fig. 1A). 1G4 CTL effectively killed NCI-H1755 target cells and moderately but significantly (*P* < .05) Mel624 cells. Recognition of antigen expressed by NCI-H1755 was also sufficient to trigger some IFNg secretion (Fig. 1B). While CTL expressing the 1G4 or MEL5 TCR could kill A375 cells to a moderate extent in some experimental repeats, such killing was indistinguishable from that by CTL not expressing a transgenic TCR and not associated with antigen-specific IFN**g** secretion (Fig. 1A, B). Endogenous antigen thus could only trigger an antigen-specific functional response of the TCR-transduced CTL when it was highly expressed.

### Agonist peptide amount and affinity and the TCR expression level control the effector function of human CTL expressing a transgenic TCR

To determine whether higher concentrations of agonist peptide can trigger more efficient TCR-mediated activation of the human CTL, we incubated the target cell lines with a high concentration, 2 µg/ml, of agonist peptide. The additional agonist peptide triggered significant (*P* < .001) increases in target cell killing in all TCR transgene/target cell combinations except for the combination of 1G4 CTL interacting with NCI-H1755 cells where killing was already efficient in the absence of exogenous peptide (Fig. 1C). Highly efficient killing was seen in at least a few experimental repeats in all TCR transgene/target cell combinations. In addition, substantial increases in IFN**g** secretion were observed (Fig. 1D). The interaction of CTL transduced to express the MEL TCR (MEL5 CTL) with target cells lacking MART-1, A375, as corroborated in the interaction of 1G4 CTL with target cells with a very low expression of NY-ESO-1, Mel624 NY-ESO-1 kd, established a low cytolysis background, around less than 2% red area change per hour, and a low background of IFN**g** secretion, around less than 500 pg/ml (Fig. 1C, D). Interaction of MEL5 CTL with the MART-1-negative A375 cells occasionally showed higher killing, up to 4% red area change per hour, but consistently low IFN**g** secretion, less than 500pg/ml (Fig. 1C, D). Thus, a background of CTL activation not driven by a transgenic TCR recognizing its cognate peptide/MHC complex is low but varies between TCRs and/or cell lines. Using a 10-fold peptide dose titration for a subset of the 1G4 CTL data, an agonist peptide concentration of 2 ng/ml was saturated for cytolysis and IFN**g** secretion (Supplementary Fig. S5A, B). We next verified that killing and IFN**g** secretion in the presence of 2µg/ml agonist peptide were TCR-dependent through comparison with nontransduced CTL (Fig. 1E, F).

We noted that IFN**g** secretion in MEL5 CTL was only moderately triggered by A375 cells incubated with the high concentration of exogenous antigenic peptide (Fig. 1D). To determine whether such moderate T-cell activation could be a consequence of a limited affinity of the TCR for the tumor-associated antigen-derived peptide/MHC complex, we used the FAT variant of the MART-1_26-35_ peptide. This variant yields a higher affinity of the MEL5 TCR for the peptide/MHC complex with a K_d_ of 3 µM as compared to 17 µM for the ELA variant [17]. Incubating A375 cells with 2 µg/ml of the FAT peptide trigger a 1.8-fold increase in cytolysis and a 7.1-fold increase in IFN**g** secretion in comparison to the ELA variant of the MART-1 peptide (Fig. 1G, H). As moderate TCR affinities for cognate peptide/MHC complexes are common in antitumor immunity, we kept mostly using the corresponding peptides here.

To determine the effects of TCR expression levels, we enhanced MEL5 TCR expression using TCR stabilization through the exchange of the human constant domains with the murine ones rather than through an additional disulfide bridge [18, 19] (Supplementary Text, Fig. S5C-D). This altered TCR stabilization increased tetramer staining of the transduced CTL from around 50% with a staining intensity barely above the background to essentially 100% with more than one log shift in intensity (Supplementary Figs. S2, S5F). IFN**g** secretion was substantially enhanced. As TCR stabilization using only an additional disulfide bridge is common in therapeutic applications, we kept using it here.

In summary, activation of primary human CTL expressing a recombinant TCR by interaction with tumor target cells could be effective yet was more often limited by low expression of tumor-associated antigens in the target cells, the only moderate affinity of the TCR for the peptide/MHC complexes and limited TCR expression when using an additional disulfide bridge to stabilize the recombinant TCR. These limitations were more severe in cytokine secretion than target cell killing and could be overcome by incubation of tumor cells with exogenous agonist peptide, by using higher affinity peptide variants, and by stabilizing the TCR with murine constant domains. Nevertheless, one of four combinations of human CTL expressing a disulfide bridge-stabilized recombinant TCR and tumor target cells tested, the interaction of 1G4 TCR-expressing CTL with NCI-H1755 target cells, allowed for the investigation of CTL activation even in response to endogenous antigen presentation.

### Primary human CTL display graded subcellular organization in response to varying antigen

The ability of CTL to form tight cell couples and maintain them in an F-actin-dependent fashion is a key cellular mechanism underpinning target cell killing and IFN**g** secretion [12, 15]. To further characterize CTL activation across stimulus strengths, we investigated two CTL tumor target cell combinations that together cover the entire stimulus range established in the functional assays, the interaction of murine constant domain-stabilized MEL5 TCR-expressing CTL (MEL5mC CTL, mCherry-labelled) with A375 cells in response to a high concentration of the high-affinity FAT peptide or no antigen (Supplementary Fig. S5D, E) and the interaction of 1G4 CTL with NY-ESO-1 wild type, plus/minus additional agonist peptide, or NY-ESO-1 knock down Mel624 cells (Supplementary Figs. S3A, 1C, D), as described sequentially here.

**Figure 2.**
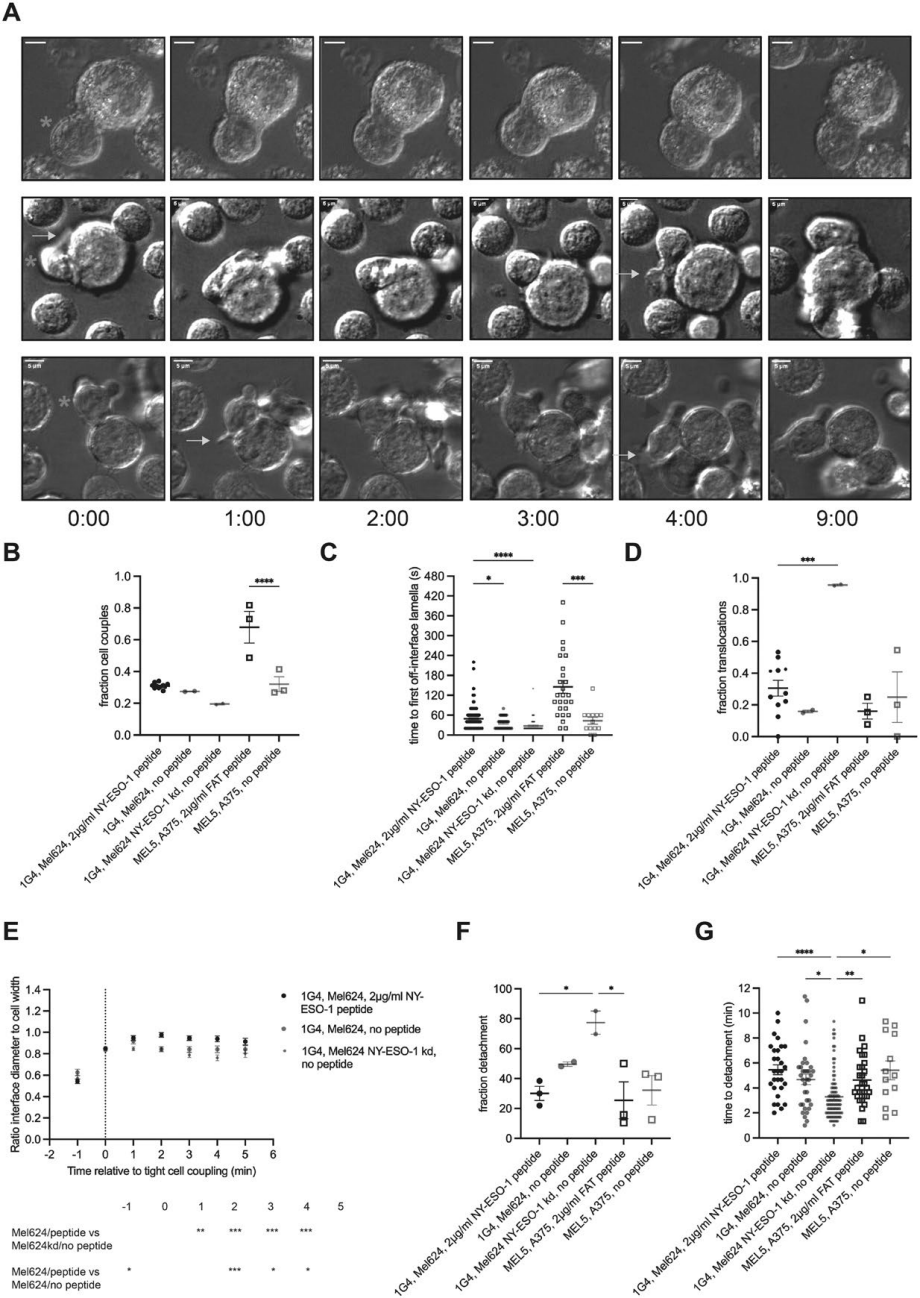
CTL morphology is regulated by stimulus strength in a graded fashion. A/ Representative bright field imaging data of the interaction between 1G4 CTL and wild type (top row) and NY-ESO-1 knockdown (middle and bottom rows) Mel624 cells in the absence of exogenous agonist peptide. Time relative to tight cell couple formation in minutes is given below the panels. The CTL is denoted with a green asterisk. The top row shows a stable cell couple, the middle row translocation, and the bottom row detachment. Off-interface lamellae are indicated with a yellow arrow, and residual attachment through the uropod with a blue triangle. One representative experiment of 2. Scale bar = 5µm. B–G. Characterization of cell morphology in the interaction of 1G4 or MEL5 CTL with Mel624, wild type (large symbol) or NY-ESO-1 knock down (small symbol), or A375 cells, respectively, in the presence of the given amount of the indicated agonist peptide as mean ± SEM. The MEL5 CTL used in these imaging experiments also expresses a chimeric costimulatory receptor in the absence of any ligand. B. Fraction of CTL converting a target cell contact into a tight cell couple. Each symbol is an imaging run. Small symbols in the MEL624 + peptide condition indicate a subset of the 1G4 CTL data matching the Mel624 and Mel624 NY-ESO-1 kd conditions. C Time from tight cell couple formation to the first off-interface lamella. Each symbol is a cell couple. D Fraction of CTL with a translocation. Small symbols in the MEL624 + peptide condition indicate a subset of the 1G4 CTL data matching the Mel624 and Mel624 NY-ESO-1 kd conditions. E Interface diameter relative to the CTL width of 1G4 CTL. Single-cell data in Supplementary Fig. S6C. MEL5 CTL data in Supplementary Fig. S6A, B. F. Fraction of CTL with detachment. Each symbol is an imaging run. G. Time from tight cell couple formation to detachment. Each symbol is a cell couple. 2–8 independent experiments. Statistical significance was determined by One-way ANOVA (B, D, F), Two-way ANOVA (E), and Kruskall-Wallis test (C, G). * *P* < .05, ** *P* < .01, *** *P* < .001, **** *P*<0.0001.

**Figure 3.**
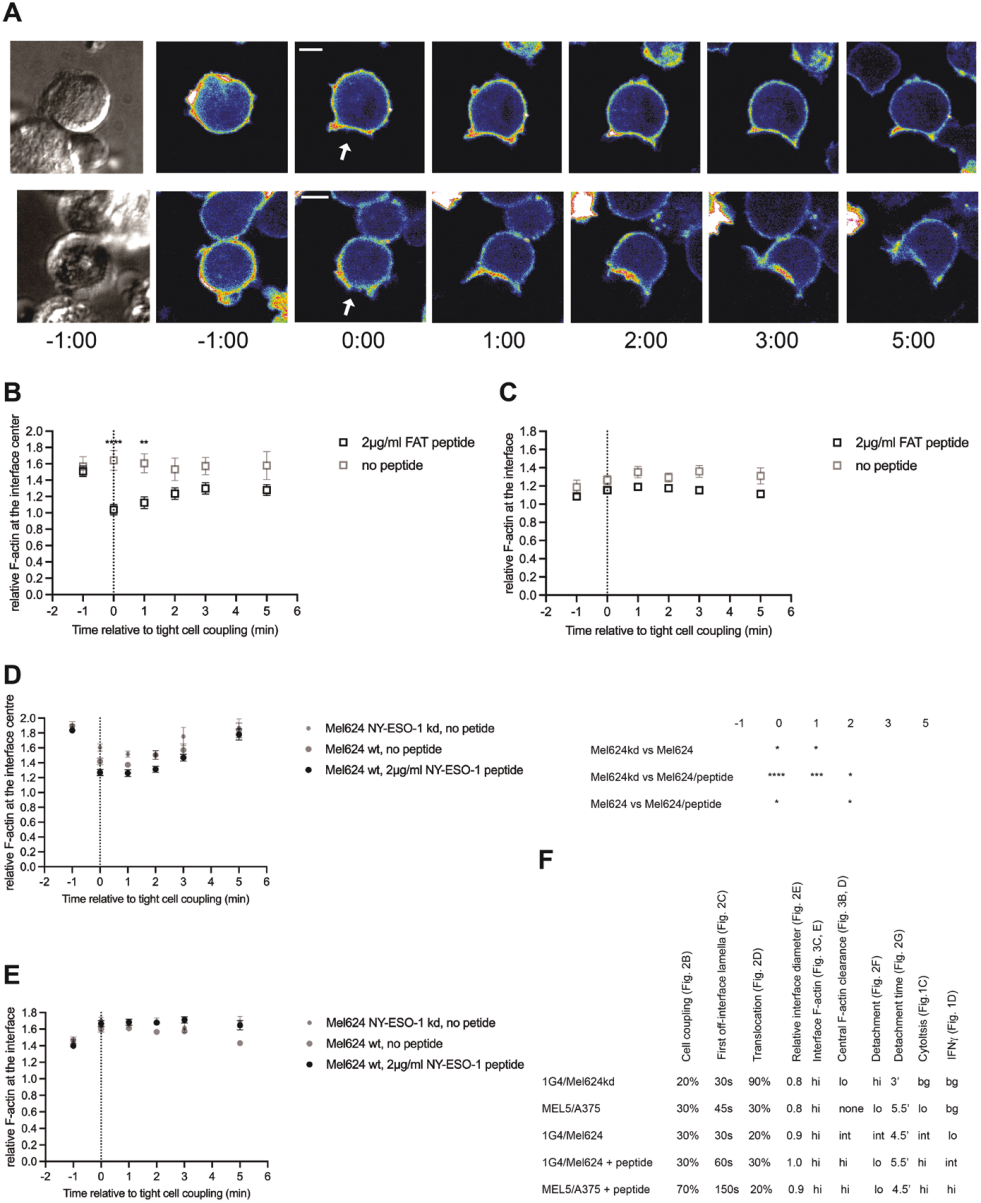
Only effective cytolysis is associated with F-actin clearance at the center of the CTL target cell interface. A. Representative spinning disk confocal imaging data of 1G4 TCR and F-tractin-GFP expressing CTL with Mel624 cells, incubated with 2 µg/ml NY-ESO-1 peptide (top) or not (bottom). At the left, a differential interference contrast image is shown at the first time point. It is followed by midplane images of F-tractin-GFP distributions at the indicated time points relative to tight cell coupling. The cellular interface is indicated with a white arrow at the time of tight cell coupling. The top row shows effective F-actin clearance at the interface center with noticeable F-actin accumulation at the interface edge, the bottom row lacks thereof. Scale bar = 5µm B, C. F-actin accumulation at the interface between CTL expressing the MEL5 TCR with F-tractin-GFP and A375 cells in the presence of the indicated amounts of agonist peptide relative to F-actin in the entire cell and to the time of tight cell coupling as mean ± SEM. Pooled data from three independent experiments. B F-actin accumulation at the central third of the interface. Single-cell data in Supplementary Fig. S7A. Statistical significance determined by Two-way ANOVA. C F-actin accumulation at the entire interface. Single-cell data in Supplementary Fig. S7B. D, E. F-actin accumulation at the interface between CTL expressing the 1G4 TCR with F-tractin-GFP and Mel624 cells, wild type or NY-ESO-1 kd, in the presence of the indicated amounts of agonist peptide relative to F-actin in the entire cell and to the time of tight cell coupling as mean ± SEM. Pooled data from 2 to 3 independent experiments. D F-actin accumulation at the central third of the interface. Single-cell data in Supplementary Fig. S7C. Statistical significance determined by Two-way ANOVA and displayed in the table to the right. E F-actin accumulation at the entire interface. Single-cell data in Supplementary Fig. S7D. F. Comparison of CTL morphological features with effector function across an increasing range of CTL stimulus strength with figures containing raw data indicated. ‘bg’ is background. * *P* < .05, ** *P* < .01, *** *P* < .001, **** *P* < .0001.

The conversion of an initial contact of a CTL with a target cell into a tight cell couple characterized by an interface as wide as the CTL is an active, F-actin-dependent process. As T cells can only form tight cell couples upon target cell contact with the leading edge [20], cell coupling frequencies of 50% and above are indicative of effective initial target cell recognition. MEL5mC CTL formed tight cell couples in 32±5% of the initial contacts with A375 target cells in the absence of antigen, less than upon addition of 2µg/ml of the FAT agonist peptide but nevertheless indicative of effective target cell recognition in the absence of antigen (Fig. 2A, B). Effective killing is associated with the maintenance of a polarized CTL target cell couple (Fig. 2C-G). Two measurements of cell couple stability are the delayed formation of lamellae pointing away from the interface and lack of translocation of the CTL over the target cell surface [12](Fig. 2A). In the absence of agonist peptide, cell couples of MEL5mC CTL with A375 target cells formed off-interface lamellae more rapidly and showed a higher frequency of translocations (Fig. 2C, D), indicative of inefficient cell couple maintenance. Interface diameters were smaller upon initial cell coupling (Supplementary Fig. S6A, B). A complete loss of polarization towards the interface, ‘detachment’, was defined as consistent leading edge lamellae at the opposite side of the CTL target cell interface (Fig. 2A). Breaking of the CTL target cell membrane contact occurred substantially later. Such detachment occurred in a minority of cell couples with no difference in frequency and time to detachment in the presence versus absence of 2 µg/ml FAT agonist peptide (Fig. 2F, G). Cell couple formation is associated with overall F-actin accumulation as imaged using F-tractin-GFP at the cellular interface [12]. The rapid clearing of F-actin at the interface center (Fig. 3A) is required for the effective progression of the cell couple to cytolysis [12, 15]. Central F-actin clearance was absent in MEL5mC CTL in the absence of antigen (Fig. 3B, Supplementary Fig. S7A), while overall interface F-actin accumulation was not impaired (Fig. 3C, Supplementary Fig. S7B). Together these data suggest that MEL5mC CTL perceives a substantial stimulus when contacting A375 target cells in the absence of antigen, triggering effective cell coupling and F-actin recruitment to the cellular interface. However, the stimulus was too weak to allow effective cell couple maintenance in preventing immediate off-interface lamellae and translocations, a full generation of a wide interface and central F-actin clearance, consistent with target cell killing at or just above background (Fig. 1C), and the lack of IFN**g** secretion (Fig. 1D).

In the interaction of 1G4 CTL with Mel624 target cells stimulus strength was varied from a high concentration of exogenous NY-ESO-1 agonist peptide to endogenous antigen expression to knock down of NY-ESO-1 expression. Cell couple formation and maintenance were highly inefficient in the interaction of 1G4 CTL with NY-ESO-1 knockdown Mel624 target cells. The cell coupling frequency was low at 19%, off-interface lamellae were instantaneous, translocation occurred in virtually all cell couples and the CTL target cell interface was narrower (Fig. 2B-E, S6C). 76±9% of 1G4 CTL cell couples with NY-ESO-1 knock down Mel624 target cells detached with a significantly (*P* < .05) shortened time to detachment of 3.3±0.2 min (Fig. 2F, G). While F-actin was effectively recruited to the cellular interface upon cell coupling, central F-actin clearance was inefficient (Fig. 3D, E, Supplementary Fig. S7C, D). Cell couples between 1G4 CTL and NY-ESO-1 knock down Mel624 target cells, while they still formed, thus were highly unstable. Whether the marginal residual cell coupling was still dependent on 1G4 TCR recognition of the NY-ESO-1 peptide/MHC complex was not resolved. Cell coupling of 1G4 CTL to wild type Mel624 cells upon endogenous antigen expression was marginally more effective than that triggered by NY-ESO-1 knockdown Mel624 cells. However, it still led to less stable cell couples than seen in the presence of a high concentration of agonist peptide, consistent with limited target cell killing and lack of IFN**g** secretion (Fig. 1C, D). While cell coupling was moderately efficient, translocations were almost completely prevented and interfaces were wider (Fig. 2B, D, E), first off-interface lamellae occurred still significantly (*P* <.05) faster than in the presence of a high concentration of agonist peptide (Fig. 2C). Central F-actin clearance showed an intermediate phenotype (Fig. 3D, Supplementary Fig. S7C). Even in the presence of exogenous antigenic peptide cell coupling between 1G4 CTL and Mel624 was only moderately efficient (Fig. 2B), suggesting that cell couple formation was less important for effector function than the subsequent cell couple maintenance. In combination with the MEL5mC CTL data, the 1G4 CTL data establish that the subcellular organization of CTL in target cell couples gradually changes from extremely unstable to fully stabilized across a wide range of stimuli strengths. Such subcellular CTL organization, more than cell couple formation per se, was related to CTL effector function (Fig. 3F).

### Interaction of primary human CTL expressing a transgenic TCR with tumor cell spheroids induces CTL suppression

In previous work, we have established that the interaction of in vitro primed murine TCR transgenic CTL with tumor cell spheroids in the presence of the cognate antigen triggers a phenotype in the spheroid-infiltrating lymphocytes (‘SIL’) that closely resembled that of suppressed tumor-infiltrating lymphocytes [12]. Key features of this phenotype are impaired effector function, upregulation of inhibitory receptor expression, a severe calcium signaling defect, and a reduced ability to maintain a fully polarized cell coupled with target cells [12]. We therefore determined whether incubation of human primary CTL expressing a transgenic TCR with tumor cell spheroids could trigger suppression.

**Figure 4.**
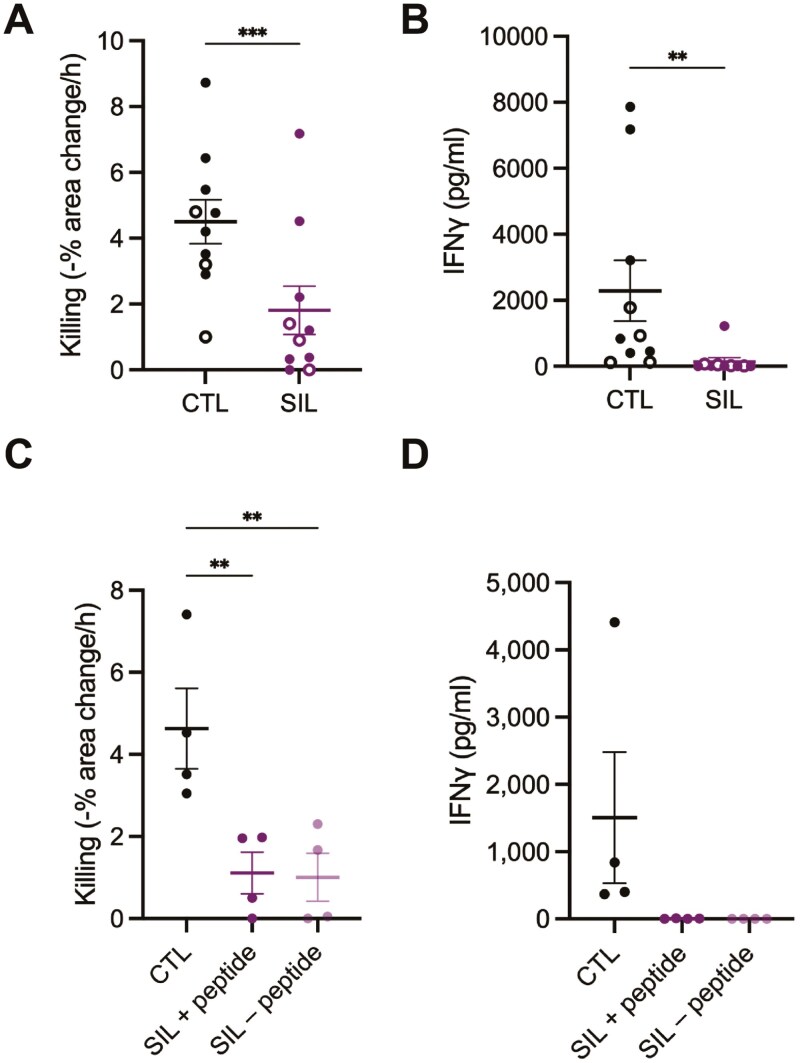
CTL interaction with spheroids induces CTL suppression. A, B. Killing of A375 (open symbols) or Mel624 (closed symbols) tumor target cells incubated with 2 µg/ml NY-ESO-1 agonist peptide by 1G4 CTL or SIL and IFNg amounts in supernatants after 16 hours interaction as mean ± SEM. Ten independent experiments. Statistical significance was determined by paired Student’s *t*-test. A. Killing, B. IFNg amounts C, D. Killing of Mel624 tumor target cells incubated with 2 µg/ml NY-ESO-1 agonist peptide by 1G4 CTL or SIL with Mel624 spheroids used in the SIL generation incubated with 2 µg/ml of exogenous NY-ESO-1 agonist peptide (‘SIL peptide’) or not (‘SIL −peptide’) and IFNg amounts in supernatants after 16 hours interaction as mean ± SEM. Four independent experiments. Statistical significance was determined by paired One-way ANOVA. C. Killing, D. IFNg amounts. ** *P* < .01, *** *P* < .001.

To allow effective suppression of CTL, tumor cell lines must be able to form stable spheroids as characterized in the Supplementary Materials (Supplementary Text, Fig. S8A-D). To induce CTL suppression, GFP^+++^ 1G4 CTL were mixed with spheroids that had been incubated with 2 µg/ml NY-ESO-1 agonist peptide for 1h and both cell types were embedded together in Matrigel and cultured for 16h in the absence of IL-2. Live cell imaging of the interaction of 1G4 CTL with spheroids confirmed rapid and deep infiltration of CTL into the spheroids (Supplementary Fig. S8E-G). Matrigel was dissolved, spheroids were washed to remove unbound CTL, and then mechanically disaggregated. Spheroid-infiltrating CTL (spheroid-infiltrating lymphocytes, ‘SIL’) were purified by fluorescence-activated cell sorting for GFP-positive cells. Comparing 1G4 SIL to CTL that were kept in tissue culture in the presence of IL-2, killing was significantly (*P* < .05) diminished by on average 40%, IFN**g** secretion by 79%, being undetectable in most experimental repeats (Fig. 4A, B). Overnight incubation of human primary CTL expressing a transgenic TCR with tumor cell spheroids presenting the cognate agonist peptide thus induced functional suppression in the CTL. Next, we investigated whether endogenous antigen presentation suffices for the induction of CTL suppression. Using the interaction of 1G4 CTL with Mel624 spheroids, the ability of SIL to kill tumor target cells and secrete IFN**g** was equally suppressed whether 2µg/ml exogenous NY-ESO-1 peptide was present in the spheroids or not (Fig. 4C, D). As most data have been acquired using SIL induction in the presence of exogenous peptides, we continue doing so hereon.

### Suppressed CTL expresses high levels of exhaustion markers

**Figure 5:**
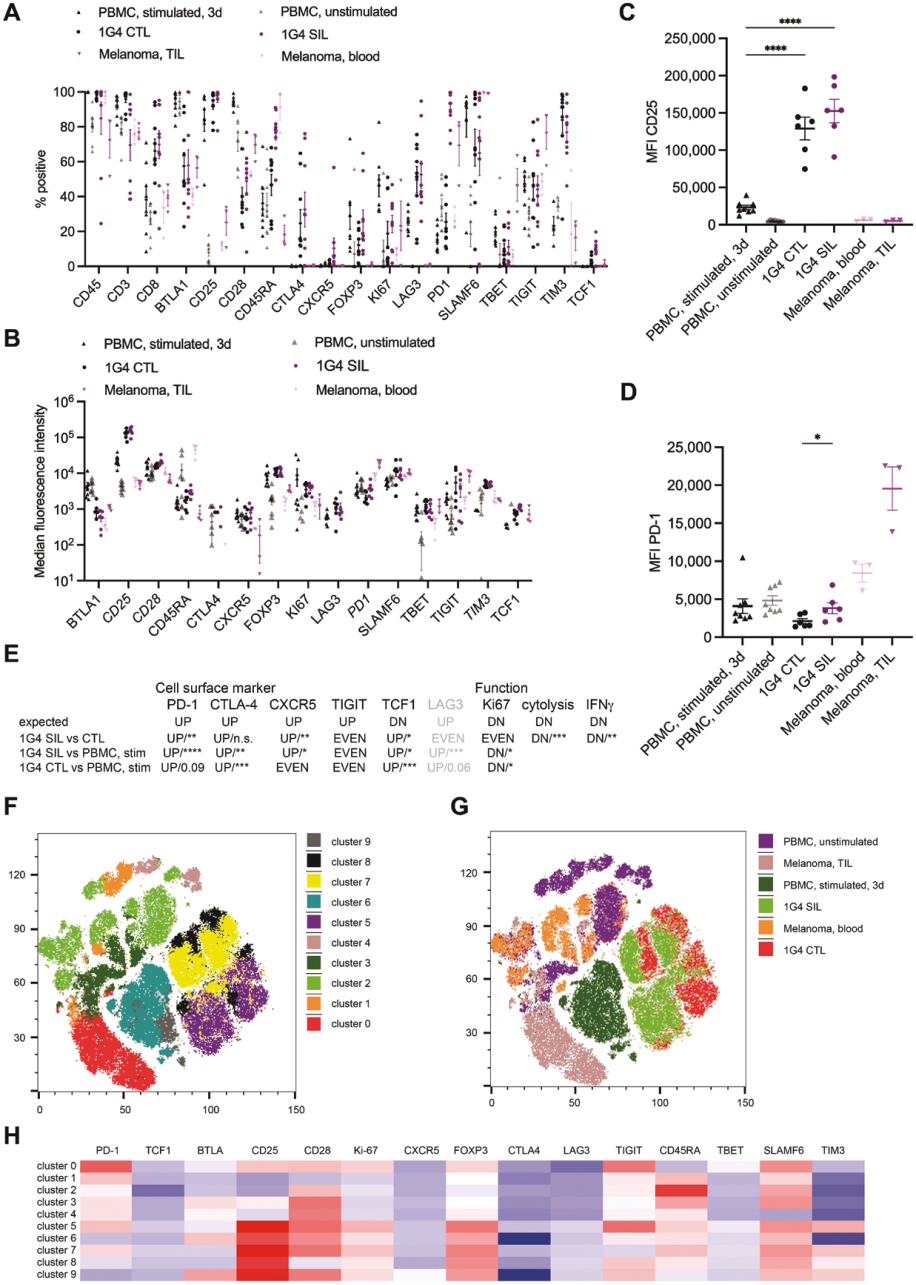
CTL interaction with spheroids induces an exhausted CTL phenotype. A–D. Percentage of CD14^–^CD19^–^CD45^+^CD3^+^CD8^+^ cells positive for the indicated markers and median fluorescence intensity as determined by flow cytometry for the indicated six experimental conditions as mean ± SEM. 3-9 independent experiments. Statistical significance was determined by One-way ANOVA for individual markers. A. Percentage cells positive. B. Median fluorescence intensity. As a default, the MFI was determined on the entire CD8^+^ T-cell population. If marker positive and negative populations could be unambiguously separated, the MFI was determined for only the positive population, as indicated by an italicized marker name. C. Median fluorescence intensity of CD25 expression for the fraction of cells gated as positive from B. D. Median fluorescence intensity of PD-1 expression for the fraction of cells gated as positive from B. Statistical significance of the difference between 1G4 CTL and SIL determined by paired Student’s *t*-test. E. Changes in cell surface marker expression (data from A) and CTL function (data from Fig. 4A, B, 5A) as ‘expected’ for exhausted CD8^+^ T cells and changes found in the comparison of the indicated cell populations. Primary exhaustion markers are in black, and secondary markers are in grey. Statistical significance was determined by Mann–Whitney *U*-test in the pairwise comparisons. F–H. Cluster analysis of the same data as in A as, F a t-SNE blot of clusters identified, G overlay of the six experimental conditions, and H expression of the indicated markers. * *P* < .05, **** *P* < .0001.

To characterize the phenotype of 1G4 CTL and 1G4 SIL, we used a 21-antibody panel with parallel detection of all antibodies on a flow cytometer equipped with a spectral analyzer. For comparison, unstimulated PBMC, PBMC stimulated with anti-CD3/CD28 beads for 72 hours, and blood and tumor-infiltrating lymphocytes from melanoma patients were also stained (Fig. 5A-E, Supplementary Fig. S9A, B). For analysis, all populations were gated for CD45^+^CD14^–^CD19^–^CD3^+^CD8^+^ cells (Supplementary Fig. S9A). 1G4 CTL were highly activated as indicated by high expression of CD25 (Fig. 5A, C) with expression of multiple inhibitory receptors, in particular LAG-3 and TIM-3, significantly (*P*<0.001) elevated in comparison to unstimulated PBMC (Fig. 5A, D, E, Supplementary Fig. S9B). Such expression was also elevated in comparison to PBMC stimulated for three days, suggesting a partial induction of exhaustion in the 1G4 CTL. 1G4 CTL in comparison to stimulated PBMC displayed a significant (*P* < .01) loss of CD28 expression (Fig. 5A) as associated with the induction of senescence [21], loss of responsiveness to persistent antigen [22] and impaired self-renewal [23]. Continuous stimulation with anti-CD3/CD28 beads over 7d in the generation of 1G4 CTL may have contributed to the partially exhausted phenotype (Supplementary Text, Fig. S9C-E).

To further characterize the phenotype of the 1G4 SIL, we compared changes in cell surface marker expression and CTL function to those expected in exhausted CD8^+^ TIL. Based on an integrated analysis of scRNAseq, epigenomics, and mass cytometry data [24, 25], 11 primary and 16 secondary cell surface markers for the distinction of exhaustion from naïve, memory, and effector CD8^+^ T cells were suggested. Five of the primary and one secondary marker were part of our antibody panel (Fig. 5E). In addition, we have analyzed T-cell proliferation, cytolysis, and IFN**g** secretion as functional markers. A comparison between PBMC stimulated for three days, 1G4 CTL and 1G4 SIL suggests a graded progression towards exhaustion. Five of seven flow cytometry-determined markers in 1G4 SIL showed an expected and significant (*P* <.05) change in comparison to three-day stimulated CD8^+^ T cells, five of nine flow cytometry and functional markers in comparison to 1G4 CTL (Fig. 5E). Two of the four markers that did not change as expected, LAG-3 and Ki67, were already up/downregulated in the 1G4 CTL compared to the three-day stimulated PMBC. The percentage of CD8^+^ T cells expressing PD-1 as a prominent activation and exhaustion marker was significantly (*P* <.0001) upregulated in 1G4 SIL, reaching levels comparable to melanoma patient TIL (Fig. 5A, Supplementary Fig. S9B), as corroborated by an analysis of median intensity (Fig 5B, D, Supplementary Fig. S9B). Thus, seven of the nine markers showed the expected progression to exhaustion, consistent with the suggestion that the 1G4 CTL has a partially and the 1G4 SIL an extensively exhausted phenotype that matches in vivo data well. The two exceptions were TIGIT where the fraction of cells expressing it did not change and TCF-1, which was upregulated in 1G4 CTL and SIL, albeit at a very low level, as further discussed below. To corroborate the single marker analysis, the flow cytometry data across all populations were clustered (Fig. 5F-H). Unstimulated PBMC and melanoma patient blood samples were well separated from the stimulated T-cell populations (Fig. 5G). Clustering confirmed the similarity between 1G4 CTL and SIL as both were distributed across clusters 5, 7, and 8 (Fig. 5F, G). Cluster 8 was enriched for 1G4 CTL over SIL and is characterized by loss of CD28 and reduced expression of multiple inhibitory receptors (Fig. 5H), suggesting a partial loss of a less active, possibly senescent 1G4 CTL population through spheroid interaction, potentially through persistent antigen exposure in the spheroids.

### Suppressed CTL displays inefficient cell couple maintenance and loss of calcium signaling

**Figure 6:**
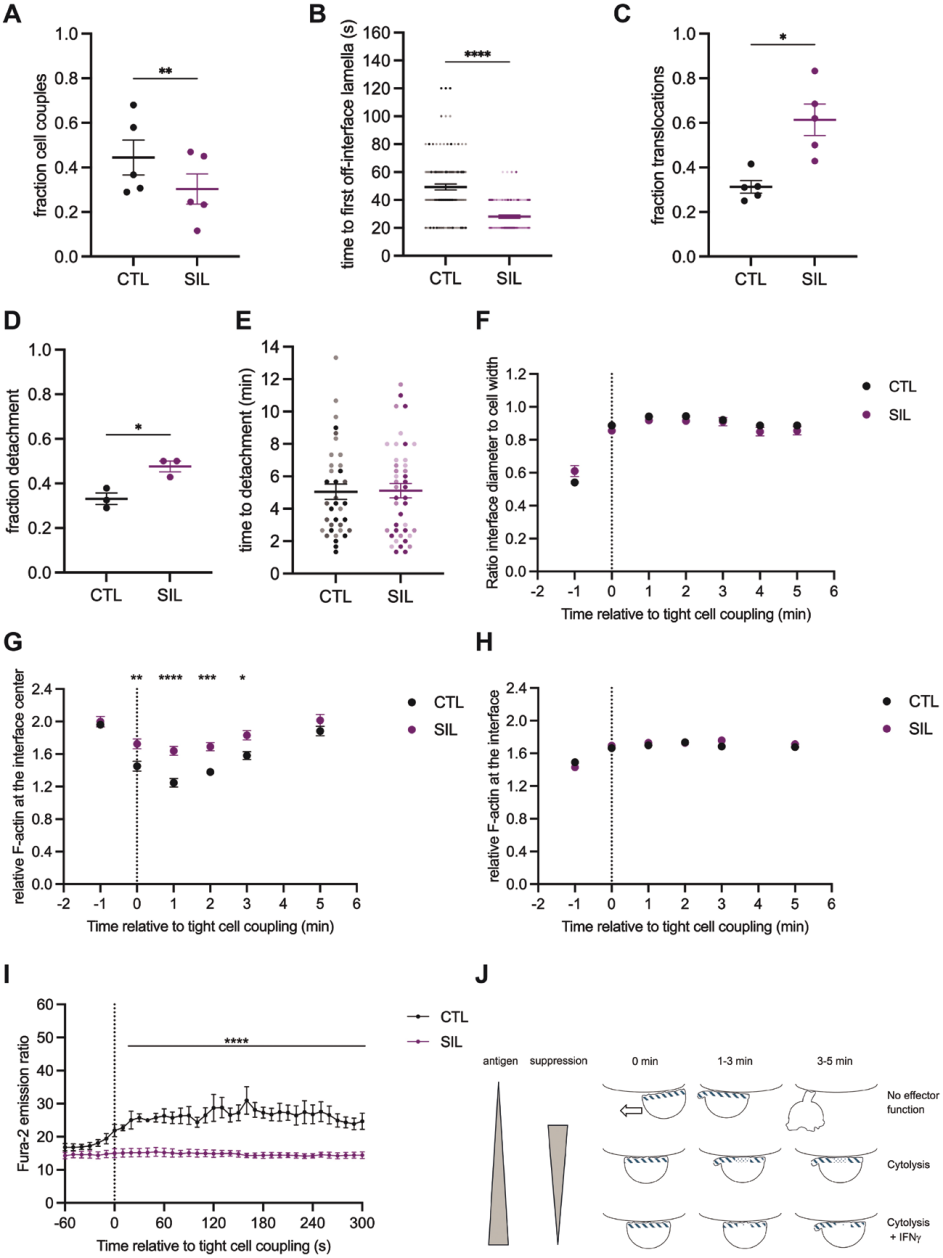
SIL tumor target cell couples are ill-maintained. A–F. Characterization of cell morphology in the interaction of 1G4 SIL or CTL with Mel624 cells in the presence of 2 µg/ml NY-ESO-1 agonist peptide as mean ± SEM. A. Fraction of T cells convert a target cell contact into a tight cell couple. Each symbol is an independent experiment. B. Time from tight cell couple formation to the first off-interface lamella. Each symbol is a cell couple. Independent experiments are marked by color intensity. C. Fraction of CTL with a translocation. Each symbol is an independent experiment. D. Fraction of CTL with detachment. Each symbol is an independent experiment. E. Time from tight cell couple formation to detachment. Each symbol is a cell couple. Independent experiments are marked by color intensity. F. Interface diameters relative to the CTL width. Single-cell data in Supplementary Fig. S10A. 3–5 independent experiments. Statistical significance was determined by paired Student’s *t*-test (A, C, D), Mann-Whitney’s *U*-test (B, E), and Two-way ANOVA (F). G, H. F-actin accumulation at the interface between SIL or CTL expressing the 1G4 TCR with F-tractin-GFP and Mel624 cells in the presence of 2 µg/ml NY-ESO-1 agonist peptide relative to F-actin in the entire cell and to the time of tight cell coupling as mean ± SEM. Pooled data from three independent experiments. G F-actin accumulation at the central third of the interface. Single-cell data in Supplementary Fig. S10B. Statistical significance determined by Two-way ANOVA. H F-actin accumulation at the entire interface. Single-cell data in Supplementary Fig. S10C. I. The Fura-2 ratio is proportional to the cytoplasmic calcium concentration of 1G4 SIL or CTL stimulated with Mel624 cells in the presence of 2 µg/ml NY-ESO-1 agonist peptide. Mean ± SEM of 4 independent experiments. Statistical significance was determined by paired Two-way ANOVA. Single-cell data in Supplementary Fig. S10D. J. Scheme of CTL subcellular reorganization in relation to antigen recognition, suppression, and effector function. CTL is binding to the target cell membrane above in each individual panel. Time is relative to tight cell couple formation. A large arrow indicates translocation, off-interface lamellae are given at the left of the CTL, if present. F-actin density at the interface and in off-interface lamellae is indicated in dark blue patterning. * *P* < 0.05, ** *P* < 0.01, *** *P* < 0.001, **** *P* < 0.0001.

The inability to maintain a stable cell coupled with an activating tumor target cell is a key characteristic of mouse tumor-infiltrating lymphocytes (TIL) [12]. We, therefore, determined the ability of 1G4 SIL to maintain stable cell couples with Mel624 target cells in the presence of 2µg/ml NY-ESO-1 agonist peptide. In comparison to 1G4 CTL, cell couples formed less efficiently (Fig. 6A) but still at a reasonably high frequency of 30±7%. First off-interface lamellae were almost instantaneous at 28±1s (Fig. 6B), the majority of cell couples showed translocations (Fig. 6C) and the frequency of detachment was increased compared to 1G4 CTL (Fig. 6D), albeit without a delay in its execution (Fig. 6E). Together these morphological defects resemble CTL activation with a limiting stimulus (Fig. 2B-D, F, G), suggesting that induction of suppression prevents CTL from maintaining stable target cell couples. In contrast to a limiting CTL stimulus (Fig. 2E, Supplementary Fig. S6A), the diameter of the SIL target cell interface was not reduced (Fig. 6F, Supplementary Fig. S10A). These data suggest that the morphological regulation of reduced SIL function while sharing many features with CTL activation upon limiting stimulation, is nevertheless distinct, as not further pursued here. The ability of 1G4 SIL to clear F-actin from the interface center was greatly impaired (Fig. 6G, Supplementary Fig. S10B), while overall interface F-actin accumulation was the same as 1G4 CTL (Fig. 6H, Supplementary Fig. S10C). Even in the presence of a strong stimulus, 2 µg/ml agonist peptide, 1G4 SIL thus were unable to maintain stable cell couples.

The elevation of the cytoplasmic calcium concentration is a key proximal T-cell signaling step. Even in response to a strong stimulus, such calcium signaling was almost entirely lost in murine TIL and SIL [12]. Similarly, when stimulated with Mel624 cells incubated with 2µg/ml NY-ESO-1 agonist peptide, 1G4 SIL did not yield detectable calcium signaling (Fig. 6I, S10D). The highly limited maintenance of 1G4 SIL cell couples together with the loss of calcium signaling are consistent with diminished 1G4 SIL effector function (Fig. 4A, B), similar to murine TIL and supports the effective induction of an exhausted phenotype (Fig. 5E).

## Discussion

### Incubation of human CTL with tumor cell spheroids induces a phenotype that closely resembles T-cell exhaustion

Incubation of human CTL expressing a transgenic TCR with antigen-presenting tumor cell spheroids induced a loss of CTL function, partially in target cell killing, almost completely in IFN**g** secretion (Fig. 4A, B). This could be caused by a reversion of CTL to a resting phenotype or by the induction of exhaustion. When CTL are deprived of IL-2 for 24h in vitro, they retain viability but display a reduction in size, indicative of reversion to a resting phenotype, and drastic changes in their proteome [26]. Two of the key changes are the loss of expression of CD25 and TIM3 by about 75%. However, CTL incubation with tumor cell spheroids led to the retention of high expression levels of CD25 and TIM3 (Fig. 5A-C), arguing against a reversion to a resting phenotype. In contrast, the expression of several markers suggested to define exhausted CTL [25] was altered as expected for the induction of CTL exhaustion (Fig. 5E). Comparing marker expression in CTL after spheroid incubation to that before and that in CTL primed for only three days in vitro to yield fully activated CTL, expression of PD-1, CTLA-4, and LAG-3 was elevated, expression of CXCR5 and Ki67 was downregulated (Fig. 5E). While the fraction of CTL expressing TIGIT was not increased (Fig. 5A), the MFI was doubled in comparison to CTL primed for only three days (Fig. 5B). All these changes are consistent with induction of CTL exhaustion upon spheroid co-culture. TCF-1 expression was slightly elevated rather than decreased, albeit at the low level of about 10% of CTL expressing TCF-1 after spheroid co-culture (Fig. 5E). As this is the frequency of TCF-1^+^ CTL seen in patients [3, 27, 28], the increase in TCF-1 expression upon spheroid co-culture likely indicates low expression of TCF-1 in the CTL to be incubated with the spheroids rather than an unusual induction of TCF-1 expression.

Comparing marker expression in the spheroid suppressed CTL to that in tumor-infiltrating CD8^+^ T cells from melanoma patients, high expression of PD-1 is shared (Fig. 5A, D, E). However, alignment with other markers such as CTLA-4 and TIM-3 is weaker. A possible explanation is that the TIL are substantially less activated as indicated by lower CD25 expression (Fig. 5A, C). While the in vitro CTL are homogeneously antigen-reactive and have thus all interacted with antigens in the spheroids, a substantial fraction of CD8^+^ T cells in tumors are bystander cells [29], likely leading to more variable activation phenotypes. With respect to the time needed to induce CTL exhaustion, exhausted CTL resides in tumors for days [30]. However, hallmarks of CTL dysfunction can be established in hours [31], as used in our spheroid experiments.

Overall, our characterization of CTL after spheroid interaction with respect to function and marker expression has established close similarity with tumor-exhausted CTL. This similarity is further corroborated by an inability of the human SIL to effectively maintain stable cell couples and a complete loss of calcium signaling (Fig. 6), a phenotype initially described in murine TIL [12]. While we can’t map the in vitro SIL in detail onto the in vivo progression from precursor to terminally exhausted TIL, the retention of some cytolytic functionality suggests that the SIL most closely resemble the intermediate bulk stage of cytolytic exhausted TIL.

### SIL as tools in therapeutic research and development

In patients, CTL acts across complex environments including the tumor microenvironment and tumor-draining lymph nodes. Clinical trials determine the efficacy of therapeutics but often struggle to elucidate their detailed mechanisms of action. For example, blocking anti-PD-1 antibodies were designed to reactivate exhausted CTL. Only years after their clinical approval it has become apparent that clonal replacement and activation of the small fraction of precursor-exhausted T cells are more likely mechanisms of action [32, 33]. A substantial number of therapeutics in the clinic and under development are T-cell targeted. These include BiTEs, ImmTACs [34, 35] and antibodies against various inhibitory and co-stimulatory receptors. An efficient determination of how such therapeutics regulate the function of suppressed CTL can serve multiple purposes. In the design of synergistic therapies, such determination can assess whether candidates have distinct mechanisms of action compared to established drugs. Using SIL, we could show that TIM-3 in contrast to PD-1 directly regulates CTL-mediated target cell killing [16]. Spheroids can be generated under the inclusion of additional cell types, e.g., cancer-associated fibroblasts. SIL generated in such spheroids allows an investigation of whether therapeutics can overcome CTL inhibition by such added cell types. Given the frequent limitations of single agents, combination therapies hold substantial promise. The scalable nature of SIL experiments should allow for more effective screening for such synergies across an extensive combinatorial space. Supporting such utility of spheroid-induced CTL suppression for the characterization of mechanisms of action of TCR-targeted therapeutics, we have in collaboration with Immunocore almost completed an investigation of how ImmTACs function in a suppressed environment in response to only endogenous amounts of antigen (Huynh *et al*., in preparation). Our findings now allow further efficient studies of more complex cellular and therapeutic combinations. Despite this utility, SIL only address CTL-targeted therapeutics in their effect on the interaction of CTL with a limited number of defined cell types, that is a part, albeit an important one of their mechanisms of action. Nevertheless, given the large number and importance of CTL-targeted drug candidates, SIL-mediated acceleration of this part of the investigation of the mechanism of action of such therapeutics should be of substantial utility.

### Spheroid properties in the induction of CTL suppression

The ability of spheroids to effectively induce CTL suppression upon co-culture raises the question which spheroid properties are key to this ability. We have not systematically investigated this question here but can offer suggestions: mechanical properties, altered metabolism, and sustained antigen presentation. Tumor cell biology changes upon growth in 3D structures. In CRISPR screens to identify cancer driver genes, hits identified in 3D spheroids matched in vivo biology more closely than hits identified in 2D tumor cell culture [36]. A key element of altered tumor cell biology is increased stiffness in a 3D environment [37]. As three-dimensional structures, spheroids force CTL to operate in a constrained environment. In vivo, biomechanical stress as sensed by CTL using Piezo1 enhances the induction of T-cell exhaustion [38]. The more constrained biomechanical environment of spheroids thus is a likely contributor to the induction of CTL suppression.

In murine renal carcinoma cells, growth in 3D spheroids alters the metabolic properties of the tumor cells. Expression of multiple amino acid transporters on the tumor cell surface is downregulated upon tumor cell growth in spheroids while expression of the glucose transporter Slc2a1 is upregulated [16], suggesting more efficient glucose uptake. Glucose competition in a 3D microenvironment can inhibit CTL effector function [39, 40]. Altered tumor cell metabolism could act directly on the CTL through metabolic competition and regulation or indirectly, as it is linked to changed biomechanics [41]. As an additional element of metabolic regulation, PD-1 engagement leads to impaired mitochondrial function as a key contributor to T-cell exhaustion [40, 42, 43]. We have shown that PD-1 is further upregulated upon CTL co-culture with spheroids (Fig. 5A, E). Altered CTL metabolism in spheroids thus is also likely to contribute to the induction of CTL suppression.

Persistent antigen exposure is a well-established inducer of CD8^+^ T-cell exhaustion [44]. However, this may not require tumor cell growth as spheroids. Here we have shown that persistent interaction of CTL with anti-CD3/CD28 beads can induce a partially suppressed CTL phenotype (Supplementary Fig. S9C-E). Small amounts of endogenously processed antigen in spheroids were as effective in inducing CTL suppression as a high concentration of exogenous agonist peptide (Fig. 4C, D), arguing that antigen amounts are not critical for the induction of suppression. These data suggest that persistent antigen presentation in spheroids alone is unlikely to be the key driver of the induction of CTL suppression. In summary, we suggest a combinatorial model of the induction of CTL suppression in spheroids were altered biomechanics and metabolism in conjunction with persistent antigen presentation and potential additional factors that we have not considered here together drive efficient induction of CTL suppression, as to be verified experimentally in the future.

### CTL effector function correlates more with the effective maintenance of target cell couples than with their formation

The execution of CTL effector function requires a series of well-characterized changes in CTL subcellular organization [15, 45–47]. The formation of a wide, F-actin-supported interface with a target cell in a sizable fraction of CTL did not require cognate antigen (Fig. 2B, 3C). While cell coupling was more efficient in the presence of antigen, about 20% of initial target cell contacts were converted to wide interfaces even in the absence of antigen. At best minimal CTL effector function was associated with such cell coupling (Fig. 1A, B). Therefore, at least part of the decision on whether to trigger the effector function must occur in the progression through the subsequent subcellular reorganization steps. Indicators of efficient CTL reorganization are the late, more than a minute, first formation of destabilizing lamellae that point away from the cellular interface with the target cell, the lack of translocation of the CTL over the target cell surface away from the initial site of binding, a wide target cell interface and a substantial reduction in F-actin content at the interface center. All four required a higher amount of agonist and needed to occur for a CTL to commit to IFNg secretion (Fig. 3F). Cytolysis was more permissive. Here rapid occurrence of the first off-interface lamellae, intermediate interface width, and only partial F-actin clearance at the interface center still allowed for substantial target cell killing. However, an almost complete absence of translocations was still required (Fig. 3F). Together, these data suggest a model of a stepwise generation of CTL effector function in response to antigen (Fig. 6J). A base level of tight cell coupling as supported by F-actin accumulation occurs even in the absence of cognate antigen and is not associated with effector function. If the CTL can remain at the site of initial cell coupling for a few minutes, even with only partial subcellular reorganization, as enabled by limiting amounts of endogenously processed antigen, cytolysis is triggered. Killing by CTL has been shown to occur on a time scale of not more than one minute [48]. Only maintenance of a fully reorganized CTL with substantial F-actin clearance at the interface center, late off-interface lamella, absence of translocations, and detachment only after several minutes requiring a larger amount of antigen allows for IFNg secretion. A less stringent antigen threshold for cytolysis versus IFNg secretion has been described before [49]. While we have focused here on antigen amounts, the role of antigen affinity on CTL subcellular organization has been extensively characterized with key roles of cell couple duration, MTOC and granule reorientation, and F-actin clearing at the interface center [45]. Together with our data here, these data are consistent with the notion that antigen affinity and amount jointly control T-cell responses [50].

In the context of this model, the induction of CTL suppression by spheroid co-culture prevents the establishment of a fully reorganized CTL even in response to high concentrations of agonist (Fig. 6). Suppressed CTL were characterized by rapid off-interface lamellae, an increased frequency of translocation and detachment as well as impaired clearance of F-actin at the interface center. According to our model, such defective subcellular reorganization is incompatible with IFNg secretion and allows only reduced cytolysis, as confirmed experimentally (Fig. 4A, B). Progressive subcellular reorganization thus offers a unified model of the regulation of CTL function where increasing antigen-mediated stimulation gradually drives more effective CTL reorganization and function while the induction of suppression counteracts it.

## Supplementary Material

ltaf023_suppl_Supplementary_Materials

## Data Availability

Raw data are available upon request.
