# Supplementary material for: Tumor cell spheroid-induced suppression of primary human cytotoxic T cells as a scalable in vitro model of exhaustion
Source: Immunother Adv. 2025 Jun 11;5(1):ltaf023. doi: 10.1093/immadv/ltaf023 (PMC12207883; doi:10.1093/immadv/ltaf023)
Supplement: ltaf023_suppl_Supplementary_Materials [file ltaf023_suppl_supplementary_materials.docx]

**Supplementary Text**

**Lentiviral TCR expression constructs**

In a human immunodeficiency virus (HIV)-derived, vesicular stomatitis virus (VSV) G protein-pseudotyped lentivirus the expression of the alpha and beta chains of a transgenic TCR and GFP as a transduction marker was driven by a spleen focus-forming virus (SFFV) promoter [1](Fig. S1A). The three proteins were linked by 2A ribosome skipping signals [2]. Furin cleavage sites allowed for the cleavage of the 2A peptides after translation. For both TCRs, pairing of the transgenic alpha and beta chains was promoted by the addition of a disulfide bridge between the constant domains [3] as used consistently here. For the MEL5 TCR we separately compared promotion of pairing of the transgenic alpha and beta chains through a disulfide bridge to that through use of murine constant domains [4] as further described below.

**NY-ESO-1 and MART-1 expression in tumor target cell lines**

Based on flow cytometry after intracellular antigen staining, all three target cell lines expressed endogenous NY-ESO-1. They did in the order of NCI-H1755 > A372 > Mel624 with 2-4-fold differences in expression between the cell lines (Fig. S3A, B). To enable investigations in the presence of even lower amounts of agonist peptide/MHC using 1G4 TCR-expressing CTL (1G4 CTL), we knocked down NY-ESO-1 in Mel624 cells (Fig. S3A-C). We noted rapid recovery of NY-ESO-1 expression in the knock down cells, possibly because of the location of the NY-ESO-1 gene on a duplicated region of the x chromosome. Therefore, we used NY-ESO-1 knock down cells only for brief periods after thawing fresh aliquots. A375 cells have previously been shown to lack MART-1 [5, 6], Mel624 cells to express it, as confirmed here (Fig. S3D) [6]. To vary antigen density, we used endogenous antigen presentation and incubation of the cell lines with a variable concentration up to 2µg/ml agonist peptide, NY-ESO-1_157-165_ and MART-1_26-35_ with the A27L mutation (‘ELA’) that enhances MHC binding [7].

**Killing of tumour target cells by CTL expressing a recombinant TCR depends on tissue culture methodology**

Lentivirally transduced CTL were cultured for 7-11 days in the presence of IL-2 plus beads coated with antibodies against CD3ε and CD28, as further explored below. Transgenic TCR expression was determined with an antibody against the Vβ element of the TCR, Vβ13.1 for the 1G4 TCR and Vβ20 for the MEL5 TCR. TCR expression was moderate, not exceeding 40% TCR Vβ-positive in CTL sorted for GFP expression of at least 10-fold above the fluorescence intensity of non-transduced CTL (‘GFP^+++^’)(Figs. S1B, C, S2A, B). The percentage of CTL transduced to express the MEL5 TCR that were positive for staining with an HLA-A*0201/MART-1 ELA tetramer was only slightly higher (Fig. S2A, B). Nevertheless, GFP^+++^ CTL could kill effectively (Fig. S1D). The cytolytic ability of GFP^+++^ CTL was rapidly declining over time of tissue culture (Fig. S1D). This was likely caused by decreasing expression of the transgenic TCR, indicated by reductions in the percentage of Vβ13.1-positive cells in the GFP^+++^ sort gate, decreased mean fluorescent intensity (MFI) of the Vβ13.1 staining, by reductions in the GFP MFI in the GFP^+++^ sort gate and a loss of blasting cell size (Fig. S1C, E-G, S2C, D). Therefore, going forward we have sorted CTL at day 7 of culture and used them the same day.

Next, we sorted CTL on day 7 of cell culture for different levels of GFP expression (Fig. S4A). Only CTL with GFP expression at least 10-fold above the fluorescence intensity of non-transduced CTL, GFP^+++^, yielded substantial cytolysis (Fig. S4B). Therefore, going forward we have used GFP^+++^ CTL. Next, we varied effector to target cell ratios in the killing assay from 1:1 to 4:1. Substantial killing occurred only at an effector to target cell ratio of 4:1 (Fig. S4C), as used going forward. In comparison, killing of neoantigen-expressing murine renal carcinoma cells by murine TCR transgenic T cells in the same imaging-based killing assay at the same high concentration of agonist peptide was already effective at an effector to target cell ratio of 1:1 [8]. Less efficient killing of the human CTL is consistent with more limited expression of the transgenic TCR in the human CTL on the background on endogenous TCR expression and/or with more limited affinity of the 1G4 and MEL5 TCRs for a tumor-associated antigen rather than a neo-antigen in the murine system as further investigated in the main manuscript part.

Human CTL were derived from blood donors such that MHC restriction of the T cells of the donor was not necessarily matched to MHC alleles expressed on the target cell lines. Moreover, the human CTL retained an endogenous TCR repertoire. Therefore, donor-to-donor variability could be expected. However, such variability was limited. Across eight donors, CTL viability after 7d of culture was above an average of 60% for each donor but one, donor 17 (Fig. S4D). Transduction efficiency for the highest GFP expression level was consistently around 20% except for donor 17 where lower viability correlated with lower transduction efficiency (Fig. S4E). Across 15 donors, there was substantial variability in killing efficiency between individual CTL cultures but not across donors (Fig. S4F). While donor-to-donor variability was moderate, we nevertheless have consistently executed experiments using cells from multiple donors because of the rare occurrence of a donor yielding CTL with substantially altered properties. Given the variability between individual CTL cultures, multiple experimental repeats were required.

**MEL5 TCR expression in CTL is enhanced upon stabilization with murine constant domains leading to increased CTL effector function**

Transgenic TCRs expressed in parallel with endogenous ones can be stabilized using an additional disulfide bridge or by exchanging the human constant domains with the murine ones [3, 4]. We used ELA and FAT peptides and A375 cells to compare CTL function upon MEL5 TCR stabilization through a disulfide bridge versus the use of murine constant domains. Primary human CTL were lentivirally transduced to express the MEL5 TCR variants (Fig. S5C) and sorted for equal expression of GFP as the transduction marker. Killing of A375 target cells was indistinguishable in the absence of agonist peptide and in the presence of 2µg/ml of the high affinity FAT peptide. Killing in response to 2µg/ml of the moderate affinity ELA peptide was more effective in CTL expressing the MEL5 TCR with the murine constant domains (Fig. S5D). In contrast to the minor effects on killing, IFNγ secretion was 39-fold and 4-fold enhanced upon MEL5 stabilization with the murine constant domains in response to 2µg/ml of the ELA and FAT peptides, respectively (Fig. S5E). A likely explanation for the enhanced function of CTL expressing the murine constant domain-stabilized MEL5 TCR was substantially increased MEL5 TCR expression as determined with tetramer staining (Fig. S5F). As TCR stabilization using only an additional disulfide bridge is common in therapeutic applications, we kept using it here.

**Formation and stability of tumor cell spheroids**

To allow effective suppression of CTL, tumor cell lines must be able to form spheroids, and such spheroids must be sufficiently stable to allow the isolation of spheroid-infiltrating CTL for subsequent analysis. Spheroid generation needed to be experimentally optimized for each tumor cell line. A375 and Mel624 spheroids were grown from single cell suspensions in Matrigel for 12 days to an average size of around 200-500µm (Fig. S8A, B) forming spherical structures (Fig. S8C). Matrigel was dissolved and spheroids were washed, a procedure required in the isolation of spheroid-infiltrating CTL. While Mel624 spheroids consistently remained intact throughout this washing procedure, A375 spheroids were falling apart in a subset of experiments. The impaired ability of A375 spheroids to form a smooth surface (Fig. S8D) may be indicative of weaker cell-to-cell adhesion, as consistent with reduced spheroid stability. NCI-H1755 cells did not form spheroids well in Matrigel. Instead, NCI-H1755 spheroids needed to be grown in agarose-coated round bottom 96 well plates in the presence of a small concentration of Matrigel, 2.5%, as to be characterized in detail elsewhere.

**Extended in vitro culture of CTL with anti-CD3/anti-CD28 beads induces a partially suppressed phenotype**

To investigate whether continuous stimulation with anti-CD3/CD28 beads over 7d in the generation of 1G4 CTL could have contributed to their partially exhausted phenotype, we set up 1G4 CTL with bead stimulation only for the first or first three days, keeping the CTL in IL-2 medium for the remainder of the culture period. Shorter stimulation led to reduced cytolytic capability (Fig. S9C), no difference in IFNγ secretion (Fig. S9D) and lower expression of PD-1 and Ki67 (Fig. S9E), consistent with a less active phenotype. Persistent 1G4 CTL stimulation thus was required for the efficient induction of cytolytic function at the expense of partial exhaustion through increased expression of inhibitory receptors.

**Supplementary Methods**

**Antibodies**

Antibodies and staining reagents used are described in the order: antigen, fluorescent label, clone, supplier, dilution/concentration, RRID:

Panel for flow cytometry

Human CD25, BV421BD, M-A251, BD Bioscience, 1:100, RRID:AB_11154578

Human CD25, BV421BD, 2A3, BD Bioscience, 1:100, RRID:AB_2738555

Human CD8α, Pacific Blue, RPA-T8 , BioLegend, 1:25, RRID:AB_493111

Human CD28, BV480, CD28.2, BD Bioscience, 1:100, RRID:AB_2739512

Human CD4, Starbright V570, RPA-T4, Bio-Rad, 1:50, RRID:AB_3099780

Human CD3ε, Super Bright 645, OKT3, eBioscience, 1:20, RRID:AB_2662368

Human CD185 (CXCR5), BV785, J252D4, BioLegend, 1:50, RRID:AB_2629527

Human CD366 (TIM-3), Super Bright 600, F38-2E2, eBioscience, 1:100, RRID:AB_26882087

Human CD45RA, R718, HI100, BD Bioscience, 1:100, RRID:AB_2916420

Human CD279 (PD-1), APC-Cy7, EH12.2H7, BioLegend, 1:100, RRID:AB_10900982

Human CD45, NovaBlue 610-30S, 2D1, eBioscience, 1:100, RRID:AB_3098021

Human CD352 (SLAMF6/Ly108), biotin, REA339, Miltenyi, 1:30, RRID:AB_2657676

Streptavidin, Star Bright Blue 675, Bio-Rad, 1:85

Human CD272 (BTLA), BB700, J168-540, BD Bioscience, 1:50, RRID:AB_2743523

Human CD152 (CTLA-4), PE-Fire 640, BNI3, BioLegend 1:20, RRID:AB_2924561

Human TIGIT, PE-vio770, REA1004, Miltenyi, 1:100, RRID:AB_2751339

Human CD223 (LAG-3), PE-Fire 810, 7H2C65, BioLegend, 1:100, RRID:AB_2927867

Human CD14, RB545, M5E2, BD Bioscience, 1:100, RRID:AB_2691229

Human CD19, RB545, SJ25C1, BD Bioscience, 1:100, RRID:AB_2688586

Human Ki-67, BV711, Ki-67, BioLegend, 1:100, RRID:AB_11218996

Human Foxp3, PE, 259D, BioLegend, 1:50, RRID:AB_492983

Human Foxp3, PE, PCH101, eBioscience, 1:50, RRID:AB_1518782

Human TCF1, Alexa Fluor 647, 7F11A10, BioLegend, 1:50, RRID:AB_2566619

Human T-bet, RB780, O4-46, BD Bioscience, 1:100, RRID:AB_2688938

Live Dead Red, Invitrogen, 1:20,000

Individual antibodies and staining reagents for flow cytometry

Human NY-ESO-1, PE, D1Q2U, Cell Signaling Technology, 1:50, RRID:AB_2799691

Human TCR Vβ20, PE, ELL1.4, Beckman Coulter, 1:30, RRID:AB_131328

Human TCR Vβ13.1, PE, IMMU222, Beckman Coulter, 1:30, RRID:AB_131326

Tetramer HLA-A*0201, MART-1_26-35_ peptide with the A27L mutation, PE, gift from L. Wooldridge (U. Bristol), 10µg/ml

For Western Blotting

Human NY-ESO-1, E978, Santa Cruz Biotechnology, 1:200, RRID:AB_784921

Human MART-1, A103, Santa Cruz Biotechnology, 1:200, RRID:AB_627912

**Lentiviral transduction and cell culture of human CTL**

Blood buffy coats were obtained from healthy donors. PBMC were isolated by density gradient centrifugation using Ficoll-Paque^TM^ (Sigma-Aldrich). These cells constitute the flow cytometry ‘PBMC, unstimulated’ sample. For the ‘PBMC, stimulated sample’ these cells were incubated with anti-CD3/CD28 beads as described below for 3d only. PMBC were cryopreserved at a concentration of 2.5x10^7^ cells/ml. To isolate CD8^+^ T cells, buffy coat cryopreserved vials were thawed, the cells were washed twice with RPMI 1640 with 10% FBS, 2mM L-glutamine, 50µM β-mercaptoethanol and resuspended in ice cold MACS buffer. CD8^+^ T cells were purified by magnetic enrichment for CD8^+^ cells using CD8 MicroBeads (Miltenyi Biotech). CD8^+^ T cells were activated using CD3/CD28 Dynabeads (Life Technologies) at bead-to-cell ratio of 1:1 in human IL-2 medium (X-VIVO 15, serum-free hematopoietic cell medium, with 2mM L-Glutamine and gentamicin (Lonza) supplemented with 5% Human AB serum (Valley Medical), 10mM neutralized N-acetyl L-Cysteine (Sigma-Aldrich), 50μM β-Mercaptoethanol (Gibco, Thermo Fisher), and 30U/ml rh-IL-2 (NIH/NCI BRB Preclinical Repository – human IL-2 medium) and incubated overnight at 37˚C and 6% CO_2_.

The 1G4 and MEL5 TCRs, as stabilized with an additional disulfide bridge in the constant domains [3], were expressed in primary human T cells using a pHR_SFFV-based lentiviral vector (RRID:Addgene_79121) with an expression cassette of alpha chain-P2A-beta chain-P2A-GFP (as a sorting marker) or F-tractin-GFP (for F-actin imaging). For the generation of lentiviral particles, HEK 293T cells (RRID:CVCL_0063; Lenti-X 293T cells, Takara) were maintained in DMEM complete medium. 1.5×10^6^ Lenti-X 293T cells were seeded in 5ml DMEM complete medium in 60x15 mm Primaria culture plates (Corning) 24h before transfection. Cells were transfected with a total of 4.5 µg plasmid DNA using Fugene HD (Promega): 0.25µg envelope vector pMD2.G (RRID:Addgene_12259), 2µg of packaging plasmid pCMV-dR8.91 (Creative Biogene), and 2.25µg of the pHR_SFFV-based transfer vector. 48h after transfection, virus containing medium was collected and filtered through a 0.45µm nylon filter. The MEL5 TCR as stabilized by swapping the human constant domains against murine ones [4] was expressed using lentiviral constructs and procedures as described in [9] with an expression cassette of either alpha chain-P2A-beta chain-E2A-GFP (functional experiments) or alpha chain-P2A-beta chain-T2A-FRα CoStAR mCherry-E2A-F-tractin-GFP (imaging experiments). FRα CoStAR mCherry is a chimeric costimulatory receptor tagged with mCherry and activated by folate receptor α expression on target cells which was absent in the imaging experiments of this manuscript. For lentiviral infection of CTL, after 24h of setting up the primary human T cell culture, 1x10^6^ T cells were mixed with 500-700µl lentivirus-containing medium in a 24-well plate medium in presence of 8µg/ml Polybrene (Sigma-Aldrich) and centrifuged for 1.5h at 2500rpm, 37 ˚C. After spinduction primary CD8^+^ T cells were resuspended in human IL-2 medium. Cells were maintained at density of less than 2x10^6^ cells/ml and if necessary spilt back to a density of 0.5-1x10^6^ cells/ml. For some experiments as indicated, CD3/CD28 Dynabeads were removed before the spinduction or two days thereafter.

**Spheroid imaging**

To determine CTL infiltration into spheroids by live cell imaging, spheroids were dissociated from Matrigel and resuspended into fresh Matrigel at a concentration of ~8 spheroids/µl. 50µl of the spheroid-Matrigel suspension was separated into Eppendorf tubes, followed by the addition of 500,000 human sorted CTL per tube. 50µl of Matrigel, containing spheroids and T cells, was plated into each well of a 24-well tissue culture plate. After Matrigel had set, 1ml of Fluorobrite medium (ThermoFisher) with 10% FBS, 2mM L-glutamine, 50µM 2-mercaptoethanol was added to each well, containing 1.5µM DRAQ7 viability dye. Images were acquired every 2h post-plating in 3µm z steps from the bottom of the spheroid to its widest point, usually 40 steps, for 12h using a Leica SP8 AOBS confocal microscope with a 10x HC PL Fluotar lens (NA=0.3). Spheroids and SIL were segmented using a custom Fiji Image J script as described before [10]. Distance of each T cell from the spheroid surface was automatically calculated using the script.

**Analysis of live cell imaging data**

Using Fiji/ImageJ [11, 12] for analysis of DIC images, tight cell couple formation was defined as the first time point at which a maximally spread immune synapse formed or 40s after initial cell contact, whichever occurred first. To assess CTL and SIL morphology in cell couples with tumor target cells, every DIC frame after tight cell couple formation was assessed for the presence of off-synapse lamellae, defined as transient membrane protrusions pointing away from the immune synapse, followed by retraction. To determine CTL translocation over the tumor cell surface, the position of the immune synapse on the tumor target cell was compared to the position at cell coupling. If the T cell had migrated by a distance greater than the diameter of the immune synapse, this was classed as translocation. The formation of a uropod that is bound to the tumor cell at the interface together with lamellae at the CTL side opposite of the interface was classed as ‘detachment’. The diameter of the CTL target cell interface was measured in the DIC image as a straight line from one end of the interface to the other. It was normalized by division through the CTL diameter, measured as a straight line across the widest part of the CTL parallel to the interface.

For analysis of F-actin distributions as imaged with F-tractin-GFP, enrichment at the interface and interface center were measured relative to F-actin across the entire cell using Fiji/ImageJ. F-tractin-GFP enrichment across the interface relative to the entire cell was determined in the maximum intensity z-projection of the GFP z-stack. The interface was defined as the 10% of the area of the T cell closest to the T cell/target cell interface. The average fluorescence intensity of the interface area and the entire cell were measured at four to six timepoints after subtracting the mean of ten background fluorescence readings. F-tractin-GFP enrichment at the centre of the interface relative to the entire cell was determined in the midplane of the GFP z-stack of the T cell of interest. The area of the interface centre was defined as the middle third of the interface as defined above, that is as 3.5% of the total cell midplane area. The average fluorescence intensity of the interface center and the entire cell were measured at four to six timepoints after subtracting the mean of ten background fluorescence readings.

For analysis of the Fura-2 imaging experiments using Fiji/ImageJ, rolling ball background fluorescence was subtracted from the fluorescence data and the ratio of the Fura-2 images upon excitation at 340nm versus 380nm was calculated and multiplied by 100 to fit into the 8-bit display scale. Average ratio within a circular region of interest of the dimensions of the T cell was determined over time for each T cell.

**Cluster analysis of flow cytometry data**

For cluster analysis, samples were gated for live, singlet, CD45^+^CD3^+^CD19^–^CD14^–^CD8^+^ cells. The FlowJo 10.10.0 clean plugin was used to reduce technical noise. Files for experimental repeats were concatenated with the FlowJo concatenation tool. CD8^+^ cells for each experimental repeat were down sampled to 18,500 cell and analyzed for CD25, CD28, CD185, CD366, CD45RA, CD279, CD352, CD272, CD152, TIGIT, CD223, Ki-67, FOXP3, TCF1, T-BET. t-SNE (t-Distribution Stochastic Neighbor Embedding) was employed to map the high-dimensional cellular data into a two-dimensional space using: iteration: 1000, perplexity: 30, K-nearest neighbors’ algorithm: Exact (vantage point tree), and gradient algorithm: Barnes-Hut. Cells were clustered by implementing FlowSOM v. 3.0.18 with 10 metaclusters and 10 × 10 grid size, all other parameters were left at the default settings.

**Supplementary References**

1. Morsut, L., et al., *Engineering Customized Cell Sensing and Response Behaviors Using Synthetic Notch Receptors.* Cell, 2016. **164**(4): p. 780-91 DOI: 10.1016/j.cell.2016.01.012.

2. Szymczak, A.L., et al., *Correction of multi-gene deficiency in vivo using a single 'self-cleaving' 2A peptide-based retroviral vector.* Nat Biotechnol, 2004. **22**(5): p. 589-94 DOI: 10.1038/nbt957.

3. Cohen, C.J., et al., *Enhanced antitumor activity of T cells engineered to express T-cell receptors with a second disulfide bond.* Cancer Res, 2007. **67**(8): p. 3898-903 DOI: 10.1158/0008-5472.CAN-06-3986.

4. Cohen, C.J., et al., *Enhanced antitumor activity of murine-human hybrid T-cell receptor (TCR) in human lymphocytes is associated with improved pairing and TCR/CD3 stability.* Cancer Res, 2006. **66**(17): p. 8878-86 DOI: 10.1158/0008-5472.CAN-06-1450.

5. Carrabba, M.G., et al., *Suboptimal activation of CD8(+) T cells by melanoma-derived altered peptide ligands: role of Melan-A/MART-1 optimized analogues.* Cancer Res, 2003. **63**(7): p. 1560-7.

6. Cormier, J.N., et al., *Natural variation of the expression of HLA and endogenous antigen modulates CTL recognition in an in vitro melanoma model.* Int J Cancer, 1999. **80**(5): p. 781-90 DOI: 10.1002/(sici)1097-0215(19990301)80:5<781::aid-ijc24>3.0.co;2-a.

7. Madura, F., et al., *TCR-induced alteration of primary MHC peptide anchor residue.* Eur J Immunol, 2019. **49**(7): p. 1052-1066 DOI: 10.1002/eji.201948085.

8. Ambler, R., et al., *PD-1 suppresses the maintenance of cell couples between cytotoxic T cells and target tumor cells within the tumor.* Sci Signal, 2020. **13**(649): p. eaau4518 DOI: 10.1126/scisignal.aau4518.

9. Kalaitsidou, M., et al., *Signaling via a CD28/CD40 chimeric costimulatory antigen receptor (CoStAR), targeting folate receptor alpha, enhances T cell activity and augments tumor reactivity of tumor infiltrating lymphocytes.* Front Immunol, 2023. **14**: p. 1256491 DOI: 10.3389/fimmu.2023.1256491.

10. Edmunds, G.L., et al., *Adenosine 2A receptor and TIM3 suppress cytolytic killing of tumor cells via cytoskeletal polarization.* Commun Biol, 2022. **5**(1): p. 9 DOI: 10.1038/s42003-021-02972-8.

11. Rueden, C.T., et al., *ImageJ2: ImageJ for the next generation of scientific image data.* BMC Bioinformatics, 2017. **18**(1): p. 529 DOI: 10.1186/s12859-017-1934-z.

12. Schindelin, J., et al., *Fiji: an open-source platform for biological-image analysis.* Nat Methods, 2012. **9**(7): p. 676-82 DOI: 10.1038/nmeth.2019.

**Supplementary Figures**

**
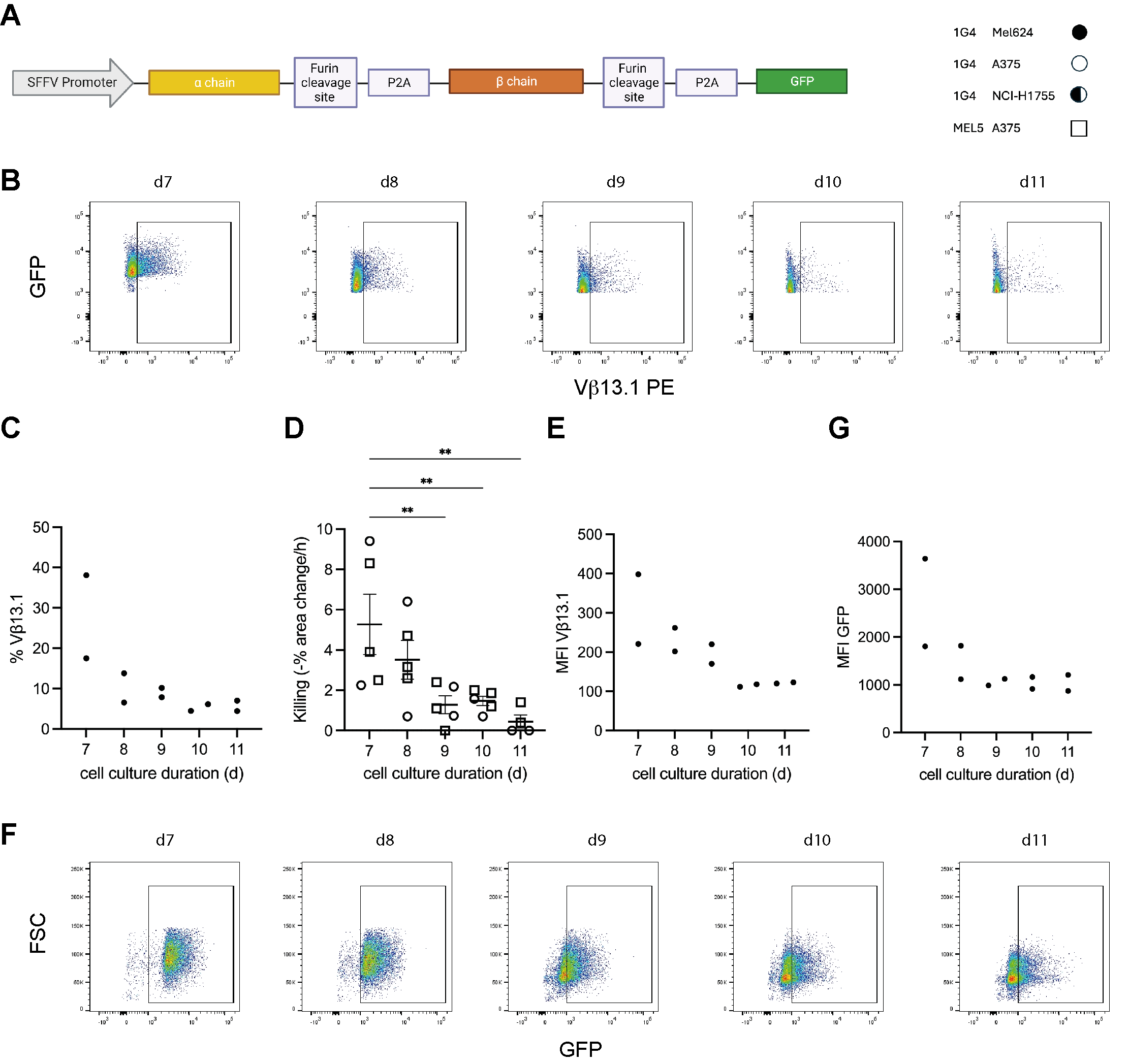
**

**Fig. S1 Transient expression of the 1G4 TCR in human primary CTL**

**A** The lentiviral cassette for the expression of the transgenic TCR and symbols used across all figures for the interaction of CTL transduced to express a transgenic TCR, 1G4 or MEL5, with indicated tumor target cell lines. **B** Representative staining data of human CTL transduced to express the 1G4 TCR with GFP as the transduction marker for the Vβ13.1 element of the 1G4 TCR after the indicated number of days of cell culture. Shown are only live cells that were sorted for high GFP expression, GFP^+++^. The gate to identify Vβ13.1-positive CTL is indicated. 1 of 2 independent experiments. **C** Percent CTL transduced to express the 1G4 TCR in the GFP^+++^ sort gate that are positive for Vβ13.1 after the indicated number of days of cell culture. 2 independent experiments. **D** Killing of A375 tumor target cells by CTL transduced to express the 1G4 or MEL5 TCR and sorted for high GFP expression after the indicated number of days of cell culture as mean ± SEM. 5 independent experiments. Statistical significance determined by paired One-way ANOVA. **E** Vβ13.1 MFI of the same cells as in C. **F** Representative GFP fluorescence data of human CTL transduced to express the 1G4 TCR with GFP as the transduction marker after the indicated number of days of cell culture. All live cells are shown. The gate to identify GFP-positive CTL is indicated. 1 of 2 independent experiments. **G** GFP MFI of the same cells as in C. 2 independent experiments. ** p<0.01.

**
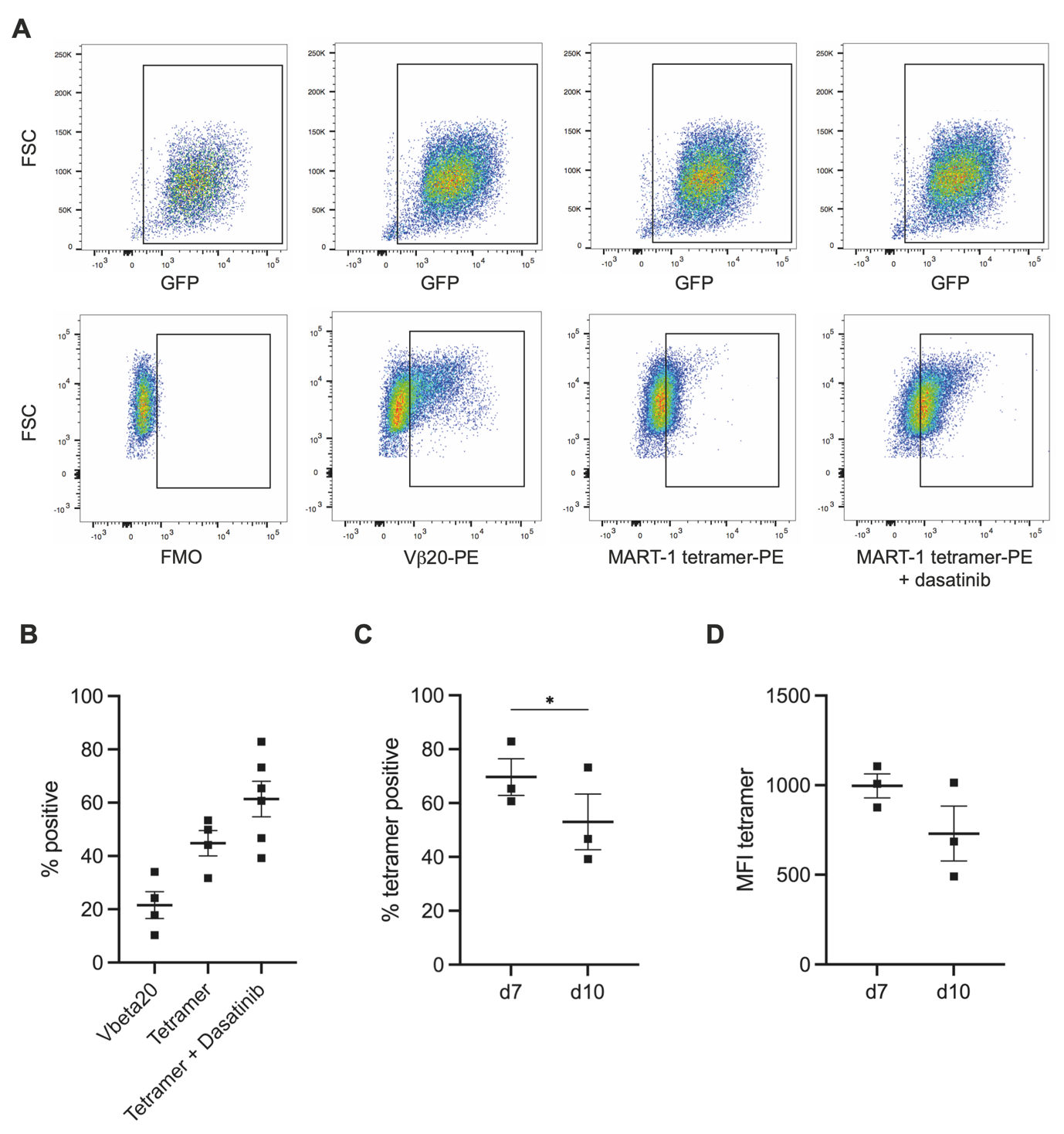
**

**Fig. S2 Transient expression of the MEL5 TCR in human primary CTL**

**A** Representative staining data of primary human CTL transduced to express the MEL5 TCR. The top row shows GFP expression as the transduction marker. The bottom row shows staining for the MEL5 TCR as indicated below each panel. Shown are all live cells. 1 of ≥4 independent experiments. **B** Percent live CTL transduced to express the MEL5 TCR on day 7 in the GFP^+++^ gate that are positive for Vβ20, the MART-1/HLA-A*0201 tetramer or the tetramer in the presence of dasatinib as mean ± SEM. 4 to 6 independent experiments. **C, D** Percent live CTL transduced to express the MEL5 TCR on day 7 and 10 in the GFP^+++^ gate that are positive for the MART-1/HLA-A*0201 tetramer in the presence of dasatinib and MFI of tetramer stain. 3 independent experiments. Statistical significance determined by paired Student’s t-test. C Percent live cells, D MFI. * p<0.05.

**
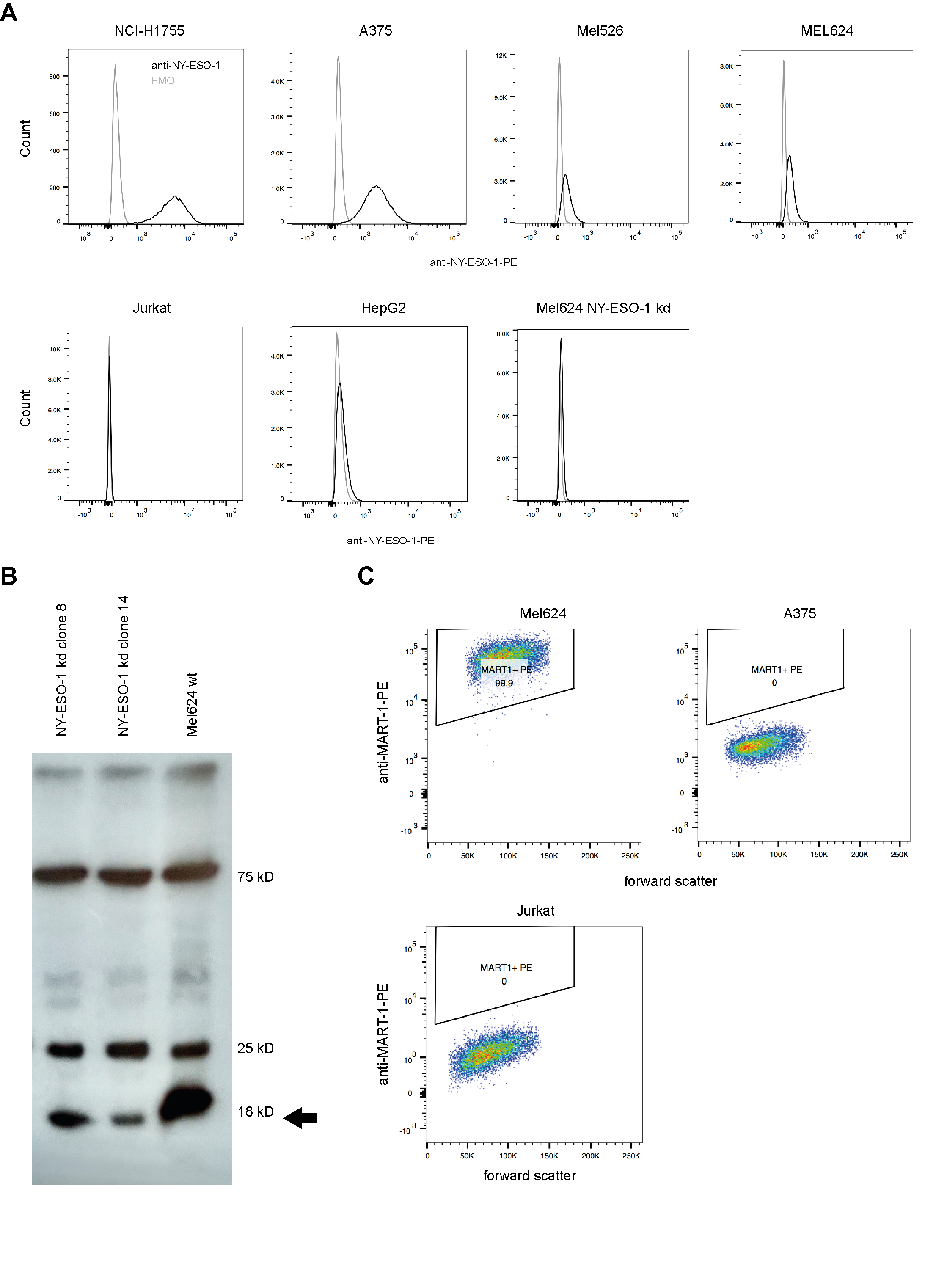
**

**Fig. S3 Expression of NY-ESO-1 and MART-1 in tumor cell lines**

**A** Quantification of flow cytometric NY-ESO-1 staining of indicated cells as log_2_ increase in MFI over fluorescence minus one (FMO) as mean ± SEM. Representative flow cytometry data in B. 1 to 4 independent experiments. Statistical significance determined by One-way ANOVA. **B** Representative staining of the indicated cells for NY-ESO-1 (black lines) in comparison to fluorescence minus one (FMO)(grey lines). 1 representative experiment of up to 4. **C** Representative Western Blot of Mel624 NY-ESO-1 knock down clones. 1 of 2 independent experiments. **D** Representative staining of the indicated cell lines for MART-1. 1 of 2 independent experiments. * p<0.05, *** p<0.001.


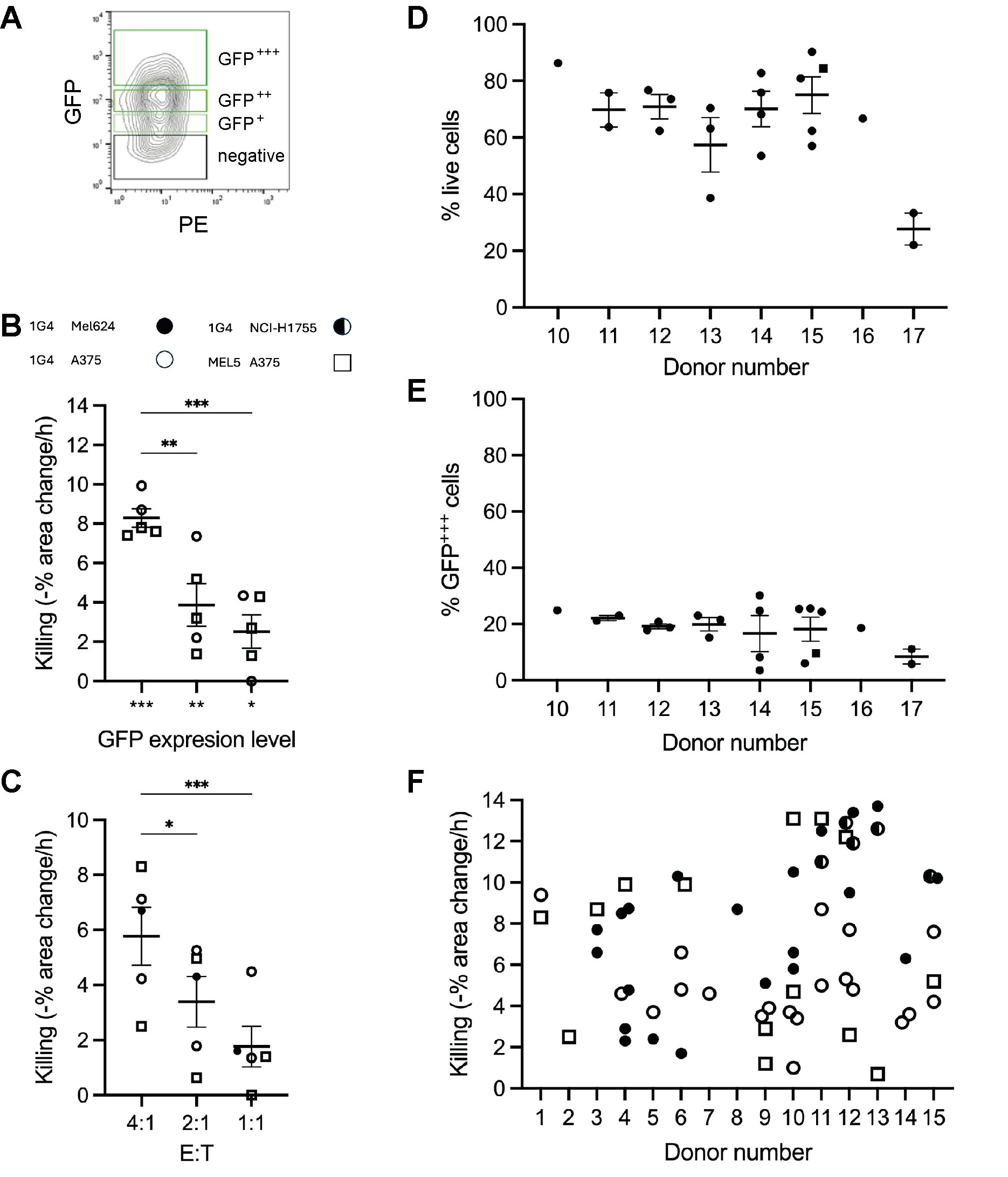


**Fig. S4 Technical parameters determining the killing of tumor target cells by primary human CTL expressing a transgenic TCR**

**A** Representative GFP sort windows of live CTL transduced to express the 1G4 TCR with GFP as the transduction marker. 1 of 5 independent experiments. **B** Killing of A375 tumor target cells by CTL transduced to express the 1G4 or MEL5 TCR and sorted for different levels of GFP expression as defined in A as mean ± SEM. 5 independent experiments. Statistical significance determined by paired One-way ANOVA. **C** Killing of A375 or Mel624 tumor target cells by CTL transduced to express the 1G4 or MEL5 TCR and sorted for GFP^+++^ expression at the indicated effector to target cell (E:T) ratios as mean ± SEM. 5 independent experiments. Statistical significance determined by paired One-way ANOVA. **D, E** Percentage of live cells (D) and of cells with GFP^+++^ expression (E) of total events for CTL transduced to express the 1G4 (circle) or MEL5 (square) TCR as mean ± SEM. 21 independent experiments. **F** Killing of A375, Mel624 or NCI-H1755 tumor target cells by CTL transduced to express the 1G4 or MEL5 TCR and sorted for GFP^+++^ expression by blood donor. 60 independent experiments. * p<0.05, ** p<0.01, *** p<0.001.

**Fig. S5 Target cell killing determined by peptide dose response and TCR stabilization through murine constant domains**

**A** Killing of A375 or Mel624 tumor target cells incubated with the indicated concentration of NY-ESO-1 agonist peptide by 1G4 CTL. 7 independent experiments. Statistical significance determined by paired Two-way ANOVA. **B** IFNγ amounts in supernatants of A375 or Mel624 tumor target cells incubated with the indicated concentration of NY-ESO-1 agonist peptide after 16h interaction with 1G4 CTL. 5 independent experiments. **C** Schematic of the two versions of the MEL5 TCR. **D, E** Killing of A375 tumor target cells, wild type (open squares) or expressing the folate receptor alpha (open squares with dot, no functional consequences in this experiment) incubated with or without 2µg/ml ELA or FAT MART-1 agonist peptide by CTL transduced to express the MEL5 TCR, stabilized with a disulfide bridge (SS) or murine constant domains (mC) as indicated and IFNγ amounts in the supernatants after 16h interaction as mean ± SEM. 6 independent experiments. Statistical significance determined by paired Two-way ANOVA. D Killing, E IFNγ amounts. **F** Tetramer staining of CTL expressing the indicated version of the MEL5 TCR. 2 independent experiments. ** p<0.01, **** p<0.0001.

**Fig. S6 CTL target cell interface diameters are regulated by stimulus strength in a graded fashion**

**A** Interface diameter relative to the CTL width of MEL5 CTL interacting with A375 cells in the presence of the given amount of the indicated agonist peptide. The MEL5 CTL used in these imaging experiments also express a chimeric costimulatory receptor in the absence of any ligand. 2 independent experiments. Single cell data in B. Statistical significance determined by Two-way ANOVA. **B** Single cell data from A. 40 (FAT peptide) and 21 (no peptide) cell couples analyzed. **C** Single cell data for Fig. 2E. On average 88 (65-101) cell couples analyzed per condition from three independent experiments. ** p<0.01

**Fig. S7 Only effective cytolysis is associated with F-actin clearance at the center of the CTL target cell interface**

Single cell data for Fig. 3B-E. Independent experiments are indicated by color intensity. On average 45 (21-60) cell couples analyzed per condition. **A** Data for Fig. 3B. **B** Data for Fig. 3C. **C** Data for Fig. 3D. **D** Data for Fig. 3E.

**Fig. S8 CTL effectively infiltrate tumor cell spheroids**

**A** Representative image of a Mel624 tumor cell spheroid. Scale bar=20µm. **B-D** Representative growth experiments of Mel625 and A375 spheroids as (B) midplane area, (C) roundness and (D) circularity as mean ± SEM. 4-19 spheroids analyzed on days 5, 7, 12 and 17. 50 spheroids analyzed on days 10 and 15. Statistical difference in circularity at days 10 and 15 determined by Student’s t-tests. **E-G** Quantification of 1G4 CTL infiltration into Mel624 or NCI-H1755 spheroids. E Representative images. 1G4 CTL express GFP, tumor cells tdTomato. F Average infiltration depth over time of 1G4 T cells interacting with spheroids as mean ± SEM. G Number of 1G4 T cells at the given infiltration depth window at the 16h time point. 9/2 independent experiments with a total of 36/6 spheroids analyzed. **** p<0.0001.

**Fig. S9 CTL interaction with spheroids induces an exhausted CTL phenotype**

**A** Sequential gating strategy for the identification of CD8^+^ T cells. **B** Representative anti-PD-1 staining data for the six experimental conditions. Only events gated as CD8α-positive are shown.1 of 3-9 independent experiments. **C, D** Killing of A375 (open symbols) or Mel624 (closed symbols) tumor target cells incubated with 2µg/ml NY-ESO-1 agonist peptide by 1G4 CTL generated in 7-day tissue culture with exposure to anti-CD3/CD28 beads for day 1 only, day 1-3 only or the entire culture period as indicated and IFNγ amounts in supernatants after 16h interaction as mean ± SEM. 10 independent experiments. Statistical significance determined by One-way ANOVA. C Killing, D IFNγ amounts. **E** Percentage of 1G4 CTL, generated in 7-day tissue culture with exposure to anti-CD3/CD28 beads for day 1-3 only or the entire culture period as indicated, positive for the indicated markers as determined by flow cytometry as mean ± SEM. 3 independent experiments. Statistical significance determined by Two-way ANOVA. * p<0.05.

**Fig. S10 SIL tumor target cell couples are ill maintained**

**A** Single cell data for Fig. 6F. Independent experiments are indicated by color intensity. 112 (CTL) and 91 (SIL) cell couples analyzed. **B, C** Single cell data for Fig. 6G, H, respectively. Independent experiments are indicated by color intensity. 77 (CTL) and 62 (SIL) cell couples analyzed. **D** Single cell data for Fig. 6I. Independent experiments are indicated by color intensity. 121 (CTL) and 41 (SIL) cell couples analyzed.
